# Supplementary figures and images for: The representation of abstract goals in working memory is supported by task-congruent neural geometry
Source: PLoS Biol. 2024 Dec 19;22(12):e3002461. doi: 10.1371/journal.pbio.3002461 (PMC11703074; doi:10.1371/journal.pbio.3002461)

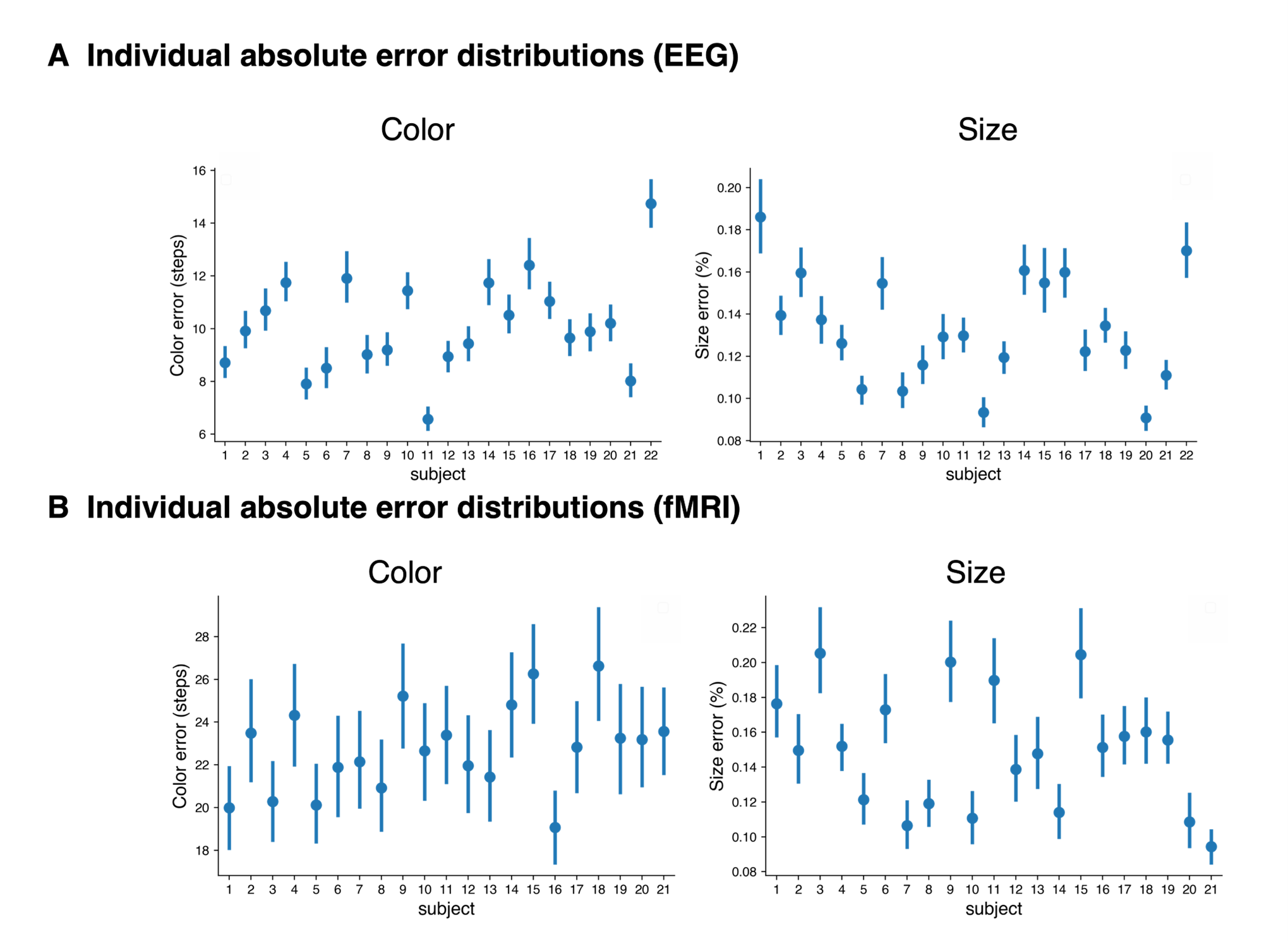

Supplement: S1 Fig — (A) Individual absolute error distributions for color (left) and size (right) responses in EEG data. Each dot represents average absolute error from individual participant. Error bar represents 95% confidence interval. (B). Same conventions as (A) but with results from fMRI data. Data and code that support these findings are available at: https://doi.org/10.57760/sciencedb.16868. (TIF) [file pbio.3002461.s001.tif]

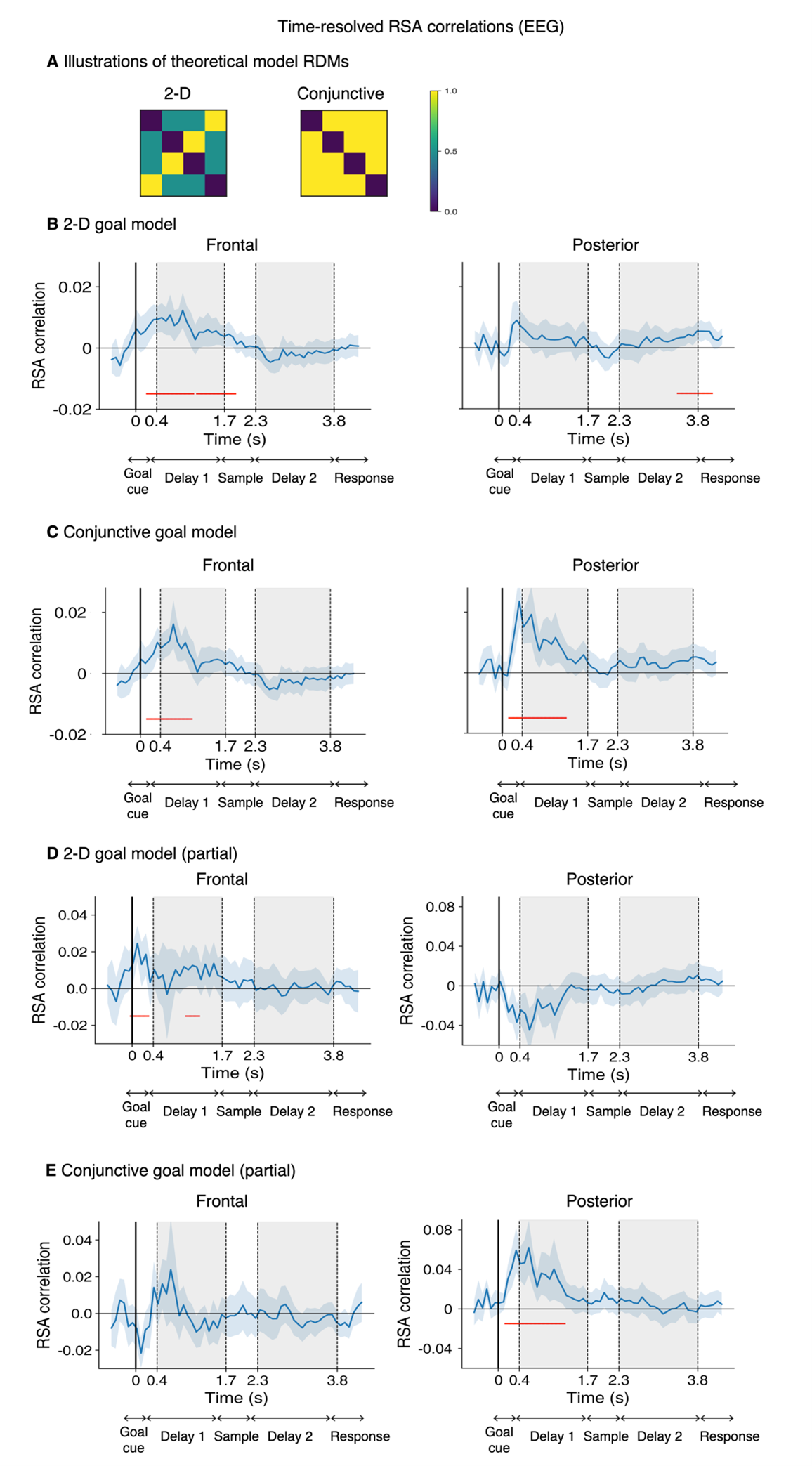

Supplement: S2 Fig — (A) Illustrations of representational distance matrices (RDMs) for 2D and conjunctive models. (B, C) Time-resolved cross-validated RSA correlations with individual data using the 2D and conjunctive goal models, estimated separately. Data was averaged temporally within a non-overlapping 80-ms sliding window. Red horizontal lines denote significant time points (α = 0.05) corrected using a cluster-based permutation test. Error bars represent 95% confidence interval. (D, E) Same as above but data-model similarities were estimated jointly in a competitive manner using partial Spearman’s rank correlation. Red horizontal lines denote significant time points without cluster-based correction. Data and code that support these findings are available at: https://doi.org/10.57760/sciencedb.16868. (TIF) [file pbio.3002461.s002.tif]

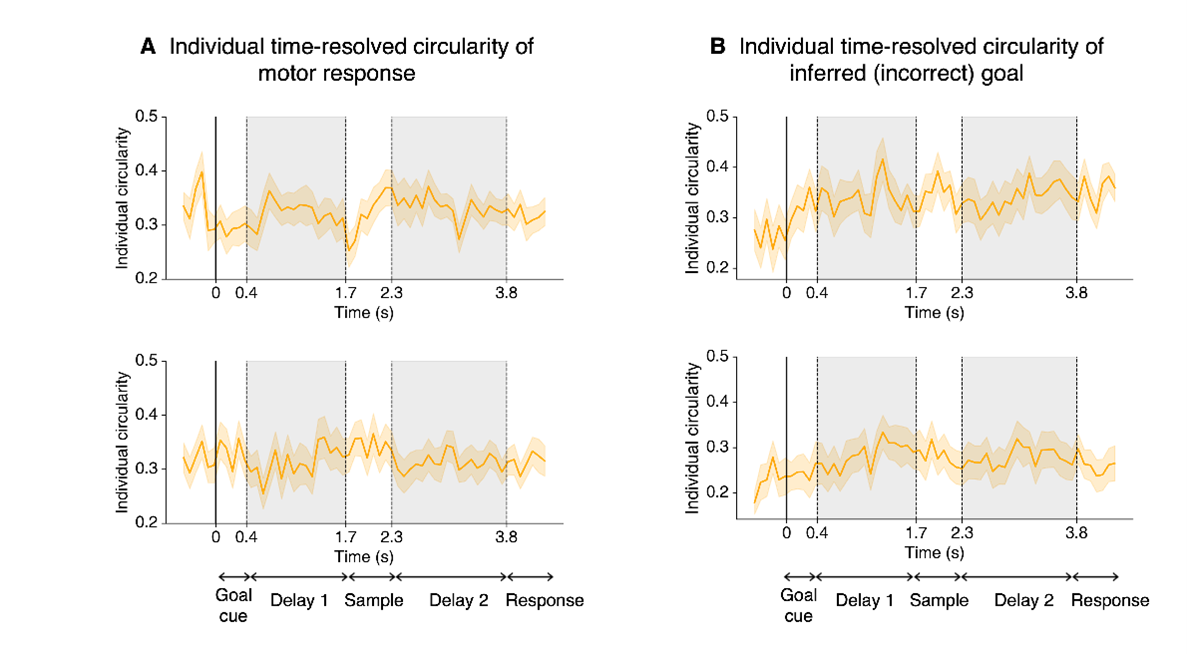

Supplement: S3 Fig — (A) Individual circularity time courses of motor response signals for frontal (upper) and posterior (lower) channels. (B) Individual circularity time courses for frontal and posterior channels using incorrect trials and condition labels, defined as those in which participants adjusted the sample stimuli to a different direction from the task goals. Error bar denotes SEM. No significant time points were found using a cluster-based permutation test. Data and code that support these findings are available at: https://doi.org/10.57760/sciencedb.16868. (TIF) [file pbio.3002461.s003.tif]

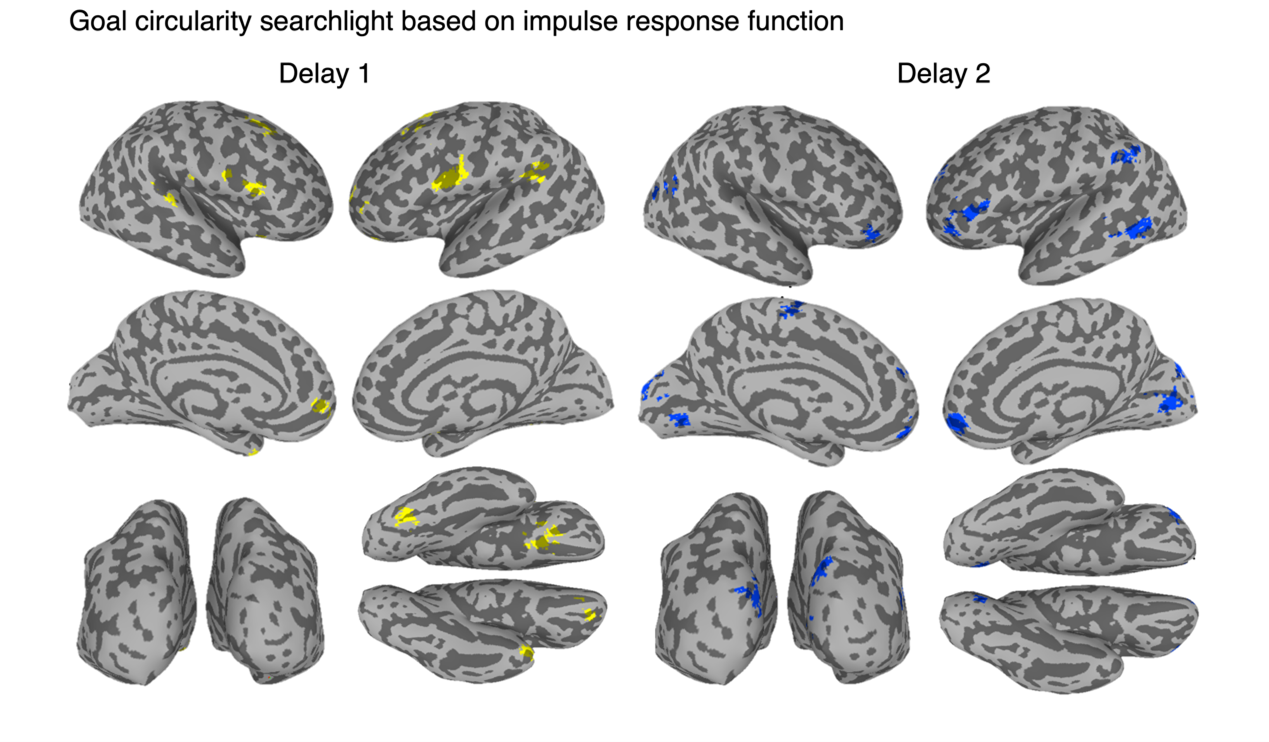

Supplement: S4 Fig — Significant ROIs showing 2D goal geometry in Delay 1 (yellow) and 2 (blue). Results were obtained by estimating delay activity using an impulse response function. Cluster-forming threshold was set to α = 0.01 and cluster-level threshold to α = 0.05 (same as the main result). Data and code that support these findings are available at: https://doi.org/10.57760/sciencedb.16868. (TIF) [file pbio.3002461.s004.tif]

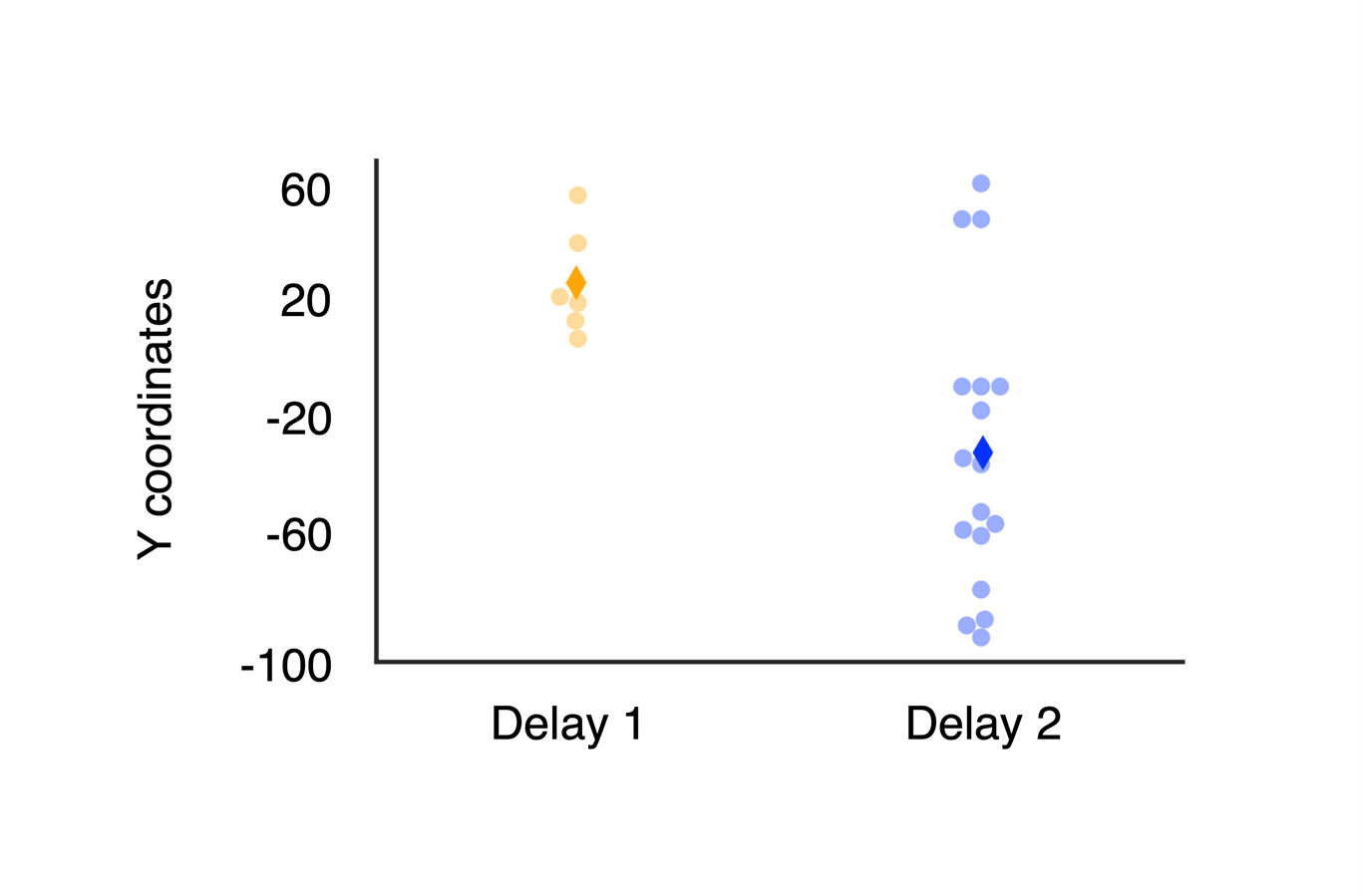

Supplement: S5 Fig — Distribution of MNI y coordinates (posterior to anterior axis) of all goal-specific clusters in the fMRI searchlight analysis. Each circle represents the center of a cluster and the diamond-shaped point represents the averaged value for each delay period. Data and code that support these findings are available at: https://doi.org/10.57760/sciencedb.16868. (TIF) [file pbio.3002461.s005.tif]

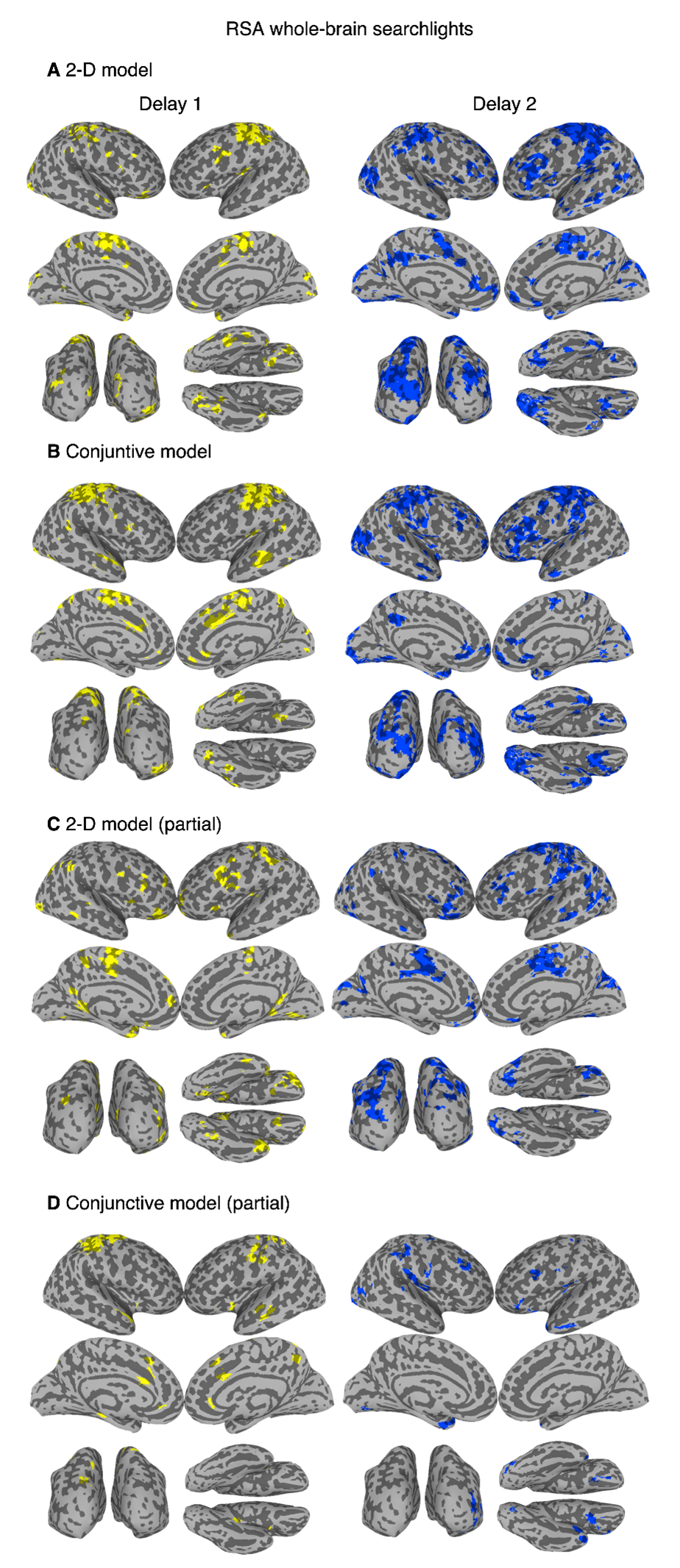

Supplement: S6 Fig — (A, B) 2D and conjunctive representations, estimated separately for each model and subjected to cluster-based correction (cluster-forming threshold = 0.05 and cluster-level threshold = 0.05). A threshold of 50 voxels was applied to all statistical maps for visualization. (C, D) Same as above but data-model similarities were estimated jointly in a competitive manner. Data and code that support these findings are available at: https://doi.org/10.57760/sciencedb.16868. (TIF) [file pbio.3002461.s006.tif]

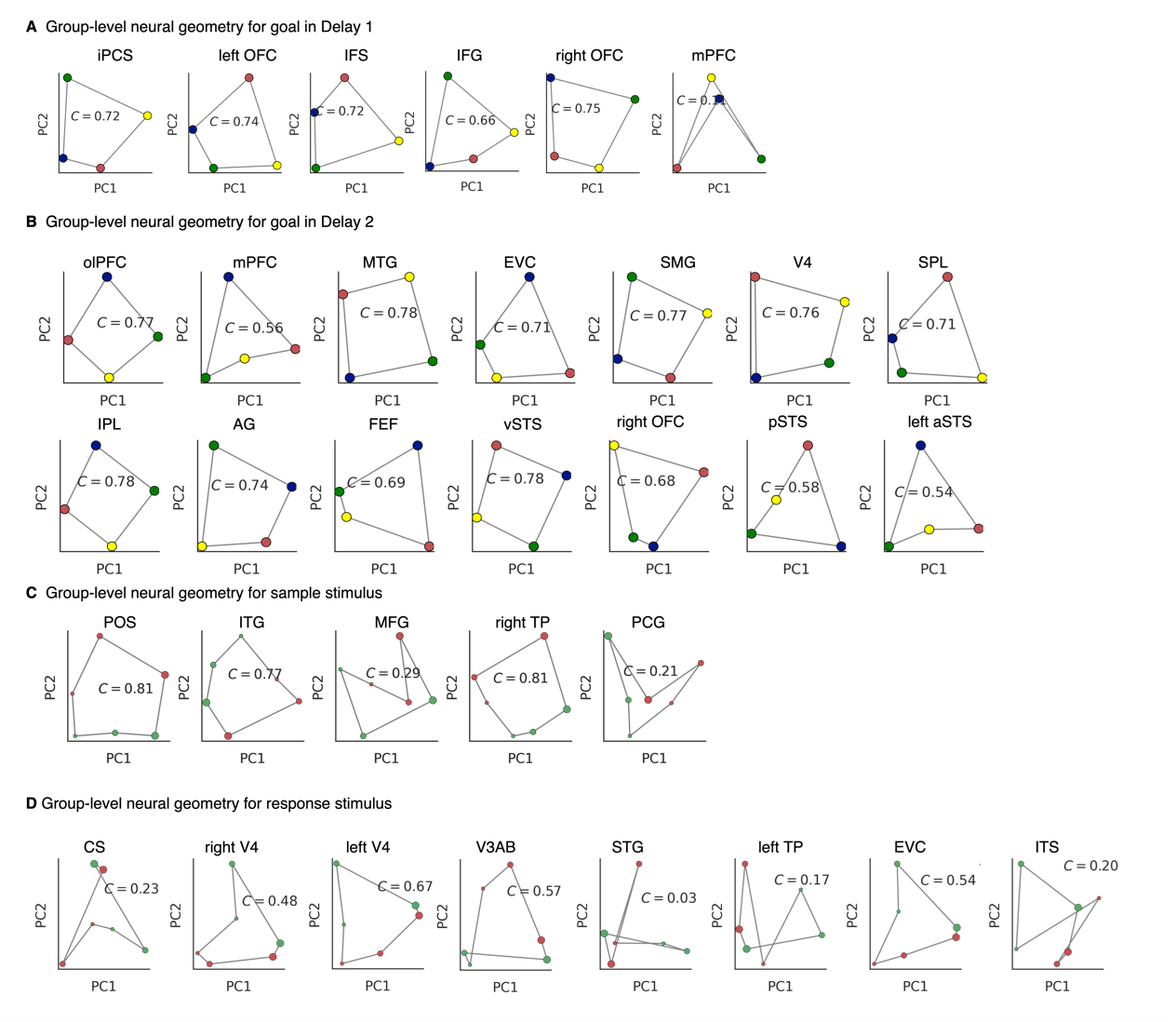

Supplement: S7 Fig — (A) Representational structures in the identified task goal subspace for Delay 1 activities. Each individual’s condition-averaged data matrix was horizontally concatenated before applying PCA. Each colored dot represented a unique condition and were connected in the same order as in the corresponding conceptual space. C denotes circularity index. (B) Same as (A) but for Delay 2. (C) Same as (A) but for sample stimulus geometry (using 6 conditions). (D) Same as (C) but for response stimulus geometry using participants’ answers as feature values. Data and code that support these findings are available at: https://doi.org/10.57760/sciencedb.16868. (TIF) [file pbio.3002461.s007.tif]

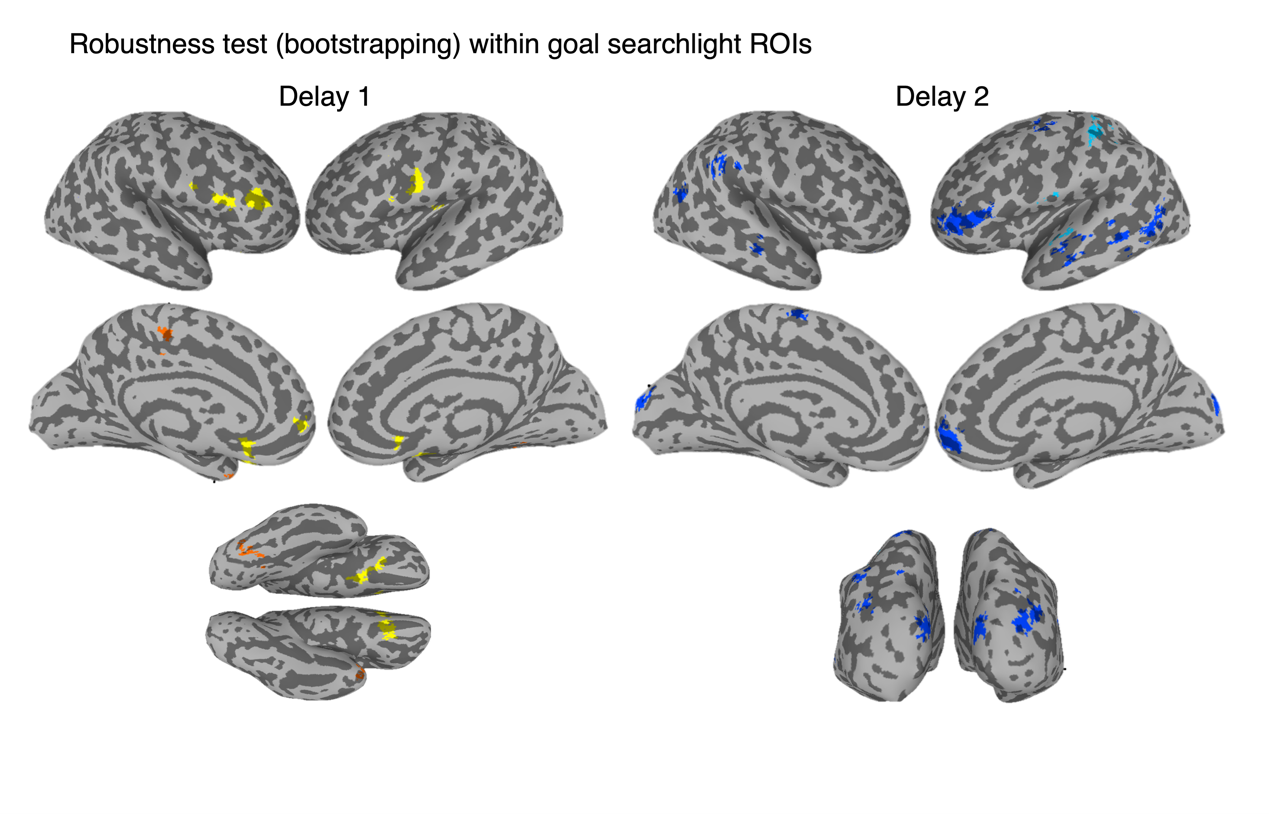

Supplement: S8 Fig — Individual circularity was repetitively calculated and averaged using trial bootstrapping within each ROI. Orange and cyan color denotes ROIs that were identified by the circularity searchlight but did not pass significance threshold in robustness test in Delay 1 and 2, respectively; yellow and blue denotes those that were significant in both searchlight analysis and robustness test (α = 0.05). Data and code that support these findings are available at: https://doi.org/10.57760/sciencedb.16868. (TIF) [file pbio.3002461.s008.tif]
