# Supplementary material for: The representation of abstract goals in working memory is supported by task-congruent neural geometry
Source: PLoS Biol. 2024 Dec 19;22(12):e3002461. doi: 10.1371/journal.pbio.3002461 (PMC11703074; doi:10.1371/journal.pbio.3002461)
Supplement: S1 Table — All p-values were FDR-corrected. (DOCX) [file pbio.3002461.s009.docx]

| **2-D geometry** | **Task epoch** | **Delay 1 delta** | | | **Delay 2 delta** | |  |
| --- | --- | --- | --- | --- | --- | --- | --- |
|  |  | *r* | *p* | *r* | | *p* | |
| **Posterior goal** | Goal cue | -0.051 | 0.617 | 0.085 | | 0.469 | |
|  | Delay 1 | 0.187 | 0.478 | 0.317 | | 0.280 | |
|  | Sample | 0.160 | 0.478 | 0.109 | | 0.469 | |
|  | Delay 2 | -0.067 | 0.617 | -0.156 | | 0.757 | |
| **Posterior stimulus** | Goal cue | 0.344 | 0.941 | 0.143 | | 0.737 | |
|  | Delay 1 | 0.248 | 0.941 | 0.134 | | 0.737 | |
|  | Sample | -0.042 | 0.850 | -0.156 | | 0.486 | |
|  | Delay 2 | -0.276 | 0.424 | -0.231 | | 0.486 | |
| **Frontal response** | Goal cue | 0.197 | 0.317 | 0.222 | | 0.213 | |
|  | Delay 1 | 0.160 | 0.317 | 0.303 | | 0.170 | |
|  | Sample | 0.019 | 0.467 | 0.134 | | 0.276 | |
|  | Delay 2 | 0.315 | 0.308 | 0.358 | | 0.170 | |
| **Posterior response** | Goal cue | 0.381 | 0.160 | 0.178 | | 0.354 | |
|  | Delay 1 | -0.009 | 0.510 | -0.032 | | 0.556 | |
|  | Sample | 0.0975 | 0.442 | 0.141 | | 0.354 | |
|  | Delay 2 | 0.147 | 0.442 | 0.140 | | 0.354 | |

**S1 Table**. Correlation of delta band coherence with 2-D geometries. All p-values were FDR-corrected.
