# Supplementary material for: The representation of abstract goals in working memory is supported by task-congruent neural geometry
Source: PLoS Biol. 2024 Dec 19;22(12):e3002461. doi: 10.1371/journal.pbio.3002461 (PMC11703074; doi:10.1371/journal.pbio.3002461)
Supplement: S2 Table — (DOCX) [file pbio.3002461.s010.docx]

| **Searchlight** | **Anatomical locations** | **Abbreviation** | **Cluster center MNI coordinates** | | |
| --- | --- | --- | --- | --- | --- |
|  |  |  | **x** | **y** | **z** |
| Stimulus circularity (Delay 2) | Parietal-occipital sulcus (left) | POS | -6 | -72 | 34 |
|  | Inferior temporal gyrus (right) | ITG | 40 | -24 | -27 |
|  | Middle frontal gyrus (left) | MFG | -25 | 60 | 17 |
|  | Temporal pole (right) | right TP | 42 | 5 | -45 |
|  | Precentral gyrus (right) | PCG | 58 | 11 | 15 |
| Response circularity (Delay 2) | Central sulcus (left) | CS | -26 | -16 | 54 |
|  | Visual area 4 (right) | right V4 | 31 | -76 | -18 |
|  | Visual area 4 (left) | left V4 | -24 | -89 | -18 |
|  | Visual area 3AB (right) | V3AB | 25 | -85 | 15 |
|  | Superior temporal gyrus (right) | STG | 53 | -7 | 1 |
|  | Temporal pole (left) | left TP | -37 | 18 | -36 |
|  | Early visual cortex (bilateral) | EVC | 5 | -94 | 9 |
|  | Inferior temporal sulcus (right) | ITS | 56 | -7 | -29 |

**S2 Table**. Result summary of whole-brain searchlight for stimulus- and response-specific 2-D geometry.
