# Supplementary material for: The representation of abstract goals in working memory is supported by task-congruent neural geometry
Source: PLoS Biol. 2024 Dec 19;22(12):e3002461. doi: 10.1371/journal.pbio.3002461 (PMC11703074; doi:10.1371/journal.pbio.3002461)
Supplement: S3 Table — (DOCX) [file pbio.3002461.s011.docx]

| Delay | Delay 1 ROI | Delay 2 ROI | Correlation | Uncorrected p-value |
| --- | --- | --- | --- | --- |
| Delay 1 | iPCS | MTG | -0.029 | 0.55 |
|  | iPCS | olPFC | 0.009 | 0.48 |
|  | iPCS | mPFC | -0.30 | 0.90 |
|  | Left OFC | MTG | -0.24 | 0.85 |
|  | Left OFC | olPFC | 0.12 | 0.30 |
|  | Left OFC | mPFC | 0.21 | 0.17 |
|  | IFS | MTG | 0.14 | 0.26 |
|  | IFS | olPFC | 0.09 | 0.34 |
|  | IFS | mPFC | -0.02 | 0.54 |
| Delay 2 | iPCS | MTG | 0.54 | 0.005 |
|  | iPCS | olPFC | -0.11 | 0.69 |
|  | iPCS | mPFC | 0.30 | 0.090 |
|  | Left OFC | MTG | -0.43 | 0.97 |
|  | Left OFC | olPFC | 0.22 | 0.15 |
|  | Left OFC | mPFC | -0.4 | 0.96 |
|  | IFS | MTG | 0.50 | 0.009 |
|  | IFS | olPFC | 0.12 | 0.29 |
|  | IFS | mPFC | 0.07 | 0.36 |

**S3 Table**. Statistics of pairwise correlation between functional connectivity and goal circularity (within Delay 2 ROI).
