# Supplementary material for: The representation of abstract goals in working memory is supported by task-congruent neural geometry
Source: PLoS Biol. 2024 Dec 19;22(12):e3002461. doi: 10.1371/journal.pbio.3002461 (PMC11703074; doi:10.1371/journal.pbio.3002461)
Supplement: S4 Table — (DOCX) [file pbio.3002461.s012.docx]

| **Delay** | **Goal-specific seed** | **Stimulus-specific seed** | **Correlation coefficient** | **p-uncorrected** | **BF10** |
| --- | --- | --- | --- | --- | --- |
| Delay 1 | iPCS | ITG | -0.66 | 0.0006 | 74.14 |
|  | left OFC | ITG | -0.43 | 0.025 | 3.20 |
|  | IFS | right V4 | -0.46 | 0.018 | 4.21 |
| Delay 2 | iPCS | POS | -0.39 | 0.040 | 2.17 |
|  | iPCS | ITG | -0.66 | 0.0006 | 76.11 |

**S4 Table**. Uncorrected statistics for correlation of functional connectivity with response error.
