# Supplementary material for: The representation of abstract goals in working memory is supported by task-congruent neural geometry
Source: PLoS Biol. 2024 Dec 19;22(12):e3002461. doi: 10.1371/journal.pbio.3002461 (PMC11703074; doi:10.1371/journal.pbio.3002461)
Supplement: Attachment — Submitted filename: ABSWM_Response letter_R1.pdf [file pbio.3002461.s013.pdf]

Point-by-point response to PBIOLOGY-D-23-03141R1

We would like to sincerely thank the reviewers for their insightful and constructive comments and suggestions, which we believe have helped us improve the manuscript substantially. In response to the reviewers' concerns, we have thoroughly edited the manuscript. Details of these changes can be found in the subsequent response to reviewers, but the key changes include:

1. Following the reviewers' suggestion, we have replaced all the group-level, fixed-effect results with results from an individual-level, random-effect approach. This method provided more stable estimates of the 2-D geometry as reported in the manuscript and further improved the rigor of this work;

2. Inspired by the reviewers' questions regarding the localization of the findings, we have adopted a searchlight approach in the fMRI study to provide a more thorough and comprehensive view of our results at the whole-brain level;

3. We have conducted additional analyses as requested by the reviewers and demonstrated that our findings cannot be accounted for by the alternative explanations;

4. We have expanded the introduction and discussion sections to better articulate our hypothesis and to elaborate on our findings.

All major changes in the revised manuscript are highlighted in red. Below we have reprinted the reviewers' comments in black, and inserted our point-by-point responses in blue.

Reviewer #1 (Runhao Lu): The manuscript describes low-dimensional neural geometry underlying abstract- (goal) and concrete- (stimulus) related representations in working memory. Using EEG and fMRI, the authors found converging evidence that the goal information first emerged in frontal areas for maintenance and then transferred to posterior areas for implementation, and the fidelity of the goal geometry is related to participants' performance. Overall, this is a neat study with lots of interesting and solid results and the manuscript on the whole is close to being publication-ready. I would happy to see the following issues answered/addressed.

We thank the reviewer for the positive assessment of our manuscript. Please see below for a detailed point-by-point response.

1. I like the method of using the state-space analysis here, but this method seems originally just suitable for single-neuron studies, in which case the 'population level' is more meaningful as it refers to multiple neurons. While in EEG/fMRI situations, they refer to multiple electrodes/voxels, which may not have a direct neurophysiological meaning. Thus, it would be nice if the authors can describe a bit more about their thoughts of using this method under the situation of EEG/fMRI and perhaps also mention its potential limitations.

Responses: We thank the reviewer for the question. Indeed, state-space analyses were originally used in the neurophysiological literature, but they have also been applied to EEG/fMRI data recently. For instance, in a recent fMRI study by Li & Curtis (Li & Curtis, 2023), a similar PCA method was used to reveal the population dynamics of spatial working memory in multiple retinotopic visual areas, including early visual, parietal, and frontal cortices. In both the Li & Curtis study and ours, we treat voxels/electrodes as the unit for population activity. The rationale is similar to that in the multivariate pattern analysis (MVPA) method. In MVPA, the idea is that, although each voxel/electrode represents aggregated activity from many neurons, the collective preference of these neurons may manifest at the single-voxel/electrode level, giving rise to decodable patterns of activity for specific information at the population level. In our case, if neurons in a group of voxels/electrodes represent the goal/stimulus structure in a two-dimensional manner, this preference may also aggregate at the voxel/electrode level, leading to the observed 2-D representational structure in the current study.

Meanwhile, we agree with the reviewer that application of state-space methods to EEG/fMRI data has its own limitations. For instance, it is possible that, although neurons in a group of voxels/electrodes represent the goal/stimulus structure in a two-dimensional manner, this population structure may fail to be detected at a coarser voxel/electrode-level. In other words, brain regions without significant goal or stimulus geometries may in fact maintain such geometries, but at a resolution undetectable by EEG or fMRI. This remains an issue for most (if not all) EEG/fMRI studies. We have included a discussion on this issue on lines 570-577 of the revised manuscript.

2. In the EEG state-space analysis, it seems a bit strange to particularly select the 3600-3800 ms time window to show the posterior geometry during late Delay 2, as it's neither pre-defined nor defined by the time-course results. Could the authors explain the reason about this choice?

Responses: To clarify, the original time window (3600-3800 ms) was indeed determined by the time course results during which the goal geometry became significant during Delay 2. We did not include the ensuing response epoch in this time window because we wanted to focus on the delay-period results in this analysis. To avoid further concerns with the selection of specific time windows, in the revised manuscript, we primarily focus on the time course results, and use epoch results only

for visualization of 2-D geometry (Figure 2 and 4). Moreover, the epochs were determined by the significant time points in the time course results. In addition, following the suggestions of Reviewers 2 and 3, we have updated all the group-level with individual-level results (see responses to Reviewer 3's Point 9 for details).

3. It seems a bit abrupt to introduce the theta coherence as a candidate mediating the backward transfer, as all previous results are based on voltage rather than frequency power/phase. I acknowledge that authors did mention theta coherence in the Introduction, but the theoretical link between theta coherence and other results still seems not very strong. Also, some more interpretation on this point in Discussion would be nice.

Responses: We thank the reviewer for raising this point. Theoretical work on working memory (and more generally on top-down processing) has proposed oscillatory coherence as a potential neural mechanism for long-range information communication between brain areas (Comeaux et al., 2023). Among different oscillatory bands, inter-areal theta-band coherence is most commonly observed in scalp EEG studies in humans, including during working memory maintenance (Daume et al., 2017; Liebe et al., 2012). The oscillatory signals can also interact with spike timing within one brain region to encode stimulus-specific information in a phase-dependent manner (Comeaux et al., 2023). Although EEG voltage differ from neuronal spiking signals, this theoretical framework nevertheless suggests a potential mechanism for how voltage signals can interact with theta oscillations. However, it's worth noting that we only reported a correlation between frontomedial theta coherence (FMT) and goal geometry in voltage signals without testing their causal relationships, and this remains a promising future avenue to explore. We have included the above information in Discussion on lines 600-606 in the revised manuscript.

4. Why the authors only focus on theta band (but not e.g. delta) as Figure 4A also showed significant effects in delta band? Also, do the authors think the backward transmission is specifically mediated by theta?

Responses: We thank the reviewer for the suggestion. Reviewer 2 also raised a similar concern (Point 4). We therefore repeated the correlation analyses using frontomedial delta (1-3 Hz) and the representational geometries, and the results are summarized below in Response Table 1 (p-values were FDR-corrected). In general, the frontomedial delta (1-3 Hz) was not related to goal or stimulus-related circularities in any epoch of the task, suggesting that the relationship between EEG oscillatory activity and representational geometry was specific to the theta band. We have added the delta band results to lines 324-326 in the revised manuscript and also to Supplementary Information (Table S1) to offer a more balanced view on the relation of EEG oscillatory activity and representational geometry.

5. The authors chose the Prefrontal and Visual ROIs for the fMRI analysis, based on the EEG results. However, posterior EEG electrodes are like to reflect both parietal and occipital activities. Considering task-relevant information is decodable throughout the frontoparietal network, I was curious about whether parietal control regions, such as the intraparietal sulcus (IPS), also contribute to the goal information coding. If possible, having some results for an IPS ROIs might be an interesting bonus.

Responses: We thank the reviewer for the suggestion. To provide a more comprehensive view of the brain regions involved in representing goal geometry in our study, we performed a whole-brain searchlight analysis for 2-D goal geometry, separately for Delay 1 and Delay 2 epochs. This whole-brain analysis revealed a cluster in Delay 2 that partially overlapped with the left IPS (Figure 6 in the revised manuscript), suggesting it may also code for the 2-D goal information. However, this region was not indicated in the subsequent functional connectivity analyses, therefore we could not further infer the function of task representation in IPS at this stage. To note, we have since replaced all ROI-based results with the new whole-brain results in the revised manuscript.

6. It is potentially very interesting that the authors found converging results for both theta phase-based connectivity and fMRI connectivity. Do the authors have any further results or discussion about their relationship or how similar they are?

Responses: Due to the distinct natures of EEG and fMRI signals, we chose to be very careful when directly comparing the results of EEG and fMRI. What we wanted to highlight in the current results are the consistent patterns that a task-related, low-dimensional goal geometry is present in both frontal channels of EEG and in subregions of frontal cortex during fMRI in the goal maintenance phase, and this goal geometry is transferred to posterior channels of EEG and posterior cortex during fMRI in the goal implementation phase. Moreover, both the frontal-to-posterior theta coherence in EEG and frontal-to-posterior functional connectivity in fMRI predicted behavioral performance and goal geometry in posterior regions, suggesting that the frontal-to-posterior connectivity patterns may mediate the goal transfer during goal implementation. On one hand, we acknowledge that these results are only correlational and further causal evidence is needed. On the other hand, without a simultaneous recording of EEG and fMRI data (as done in animal work), it would be difficult to draw a concrete conclusion on the similarity of the underlying neural signals. We leave these questions for future research, but nevertheless emphasize the functional similarities between the two modalities without directly aligning their neural basis.

Minor:

1. In the first paragraph, it would be nice to clarify/define what is 'abstract task information'.

Responses: Abstract task information refers to task contingencies, contexts, rules information, etc. We have clarified this information in the introduction.

2. Add references for the sentence starting from line 58: 'These distributed task representations are enhanced during goal implementation…'

Responses: The reference is the same as that in the next sentence and we have edited it.

3. It should be with cautious to use 'no evidence' (e.g. line 337) for the results with $p > 0.05$, given the authors were not using Bayes factors.

Responses: We have edited the wording in the sentence.

Reviewer: 2

In this work, the authors examined the nature of neural signals during a working memory task. The task was fairly complicated with a 2x2 task rule involving two features: size and color. Subjects need to update a target stimulus with the task rule to make an adjustment response. Neural data (both EEG and fMRI) were analyzed for both the rule cue and the stimulus maintenance, focusing on low-dimensional representational geometry. The main findings are that low-dimensional signals first arise in frontal sites and then moved to posterior sites; frontal-posterior interaction (esp. via theta coherence) appears to mediate such transfer; and that these signals correlate with behavior. Overall these results show how abstract goals are represented in the brain and how they are communicated among brain regions and their behavioral relevance.

Overall I find this work quite novel and exciting. It has novel analyses and interesting results. There are also a lot of results, not the least of which were two independent experiments (EEG and fMRI). It is somewhat rare in my estimate to have such amount of data in a single paper. I commend the authors for this work. At the same time, I do have some concerns about the logic, the analysis method, and the interpretation, which I think the authors should address to solidify this work.

We thank the reviewer for the positive assessment of our manuscript. Please see below for a detailed point-by-point response.

Major comments:

1. I find myself wanting to have a more intuitive explanation of what this main analysis looking for low-dimensional structure really means. Operationally, it is straightforward in that it seems to be looking for a neural replica of the factorial experimental design.

Conceptually, does this mean that only neural signals that conform to such a geometry is meaningful? This approach seems to assume that the brain operates according to how an experiment is designed. Maybe this is a reasonable idea, but it also seems a bit circular: the researchers designed a low-dimensional factorial experiment, then they looked for neural signals that reflects such geometry, then concluded the low-dimensional signal underlies the behavior (e.g., the title of the paper). Are there alternative possibilities? For one thing, does this mean that there is no higher-dimensional information or is that not captured by this method? There is some text that seems to suggest the latter, a more explicit discussion of the logic and potential limitations would be helpful.

Responses: We thank the reviewer for prompting us to explain more thoroughly the logic behind our task design and geometry analyses. In our view, the main advantage of having an experiment design with 2 orthogonal factors (task goals) is that it enables the characterization of task-related representational geometry of goal information, beyond simple decoding of the presence of goal representation without specifying its representational format. However, although the neural geometry identified in our analyses aligns well with the task structure, it is not mutually exclusive with other formats of signal. In fact, other formats such as conjunctive representation could also exist in different brain regions or at different times of the task, but these signals may not reflect the task structure and could serve other purposes. To illustrate this, we performed an RSA analysis using a conjunctive model (which assumes that all goals are equidistant without forming a 2-D structure) and compared it with the 2-D model in the main text. Interestingly, we found that the conjunctive model was significant in the posterior channels during goal presentation and maintenance (Goal cue and Delay 1). By contrast, the 2-D model was significant during Delay 2 towards Response, consistent with our circularity analysis (Response Figure 1). These results suggest that sensory representation of goals is more likely to be in the conjunctive format (as it lacks a specific structure), while the implementation of goals is more likely to be in the 2-D format, in line with our proposal that low-dimensional geometry underlies goal transfer and implementation. We have made clearer in the revised manuscript (on lines 110-129; 175-177; 499-503) the reasoning of our experimental design. We also believe that it would be very interesting in future studies to include goal structures of different dimensionalities, which could serve as direct comparison and will add to a more comprehensive view of the goal geometry.

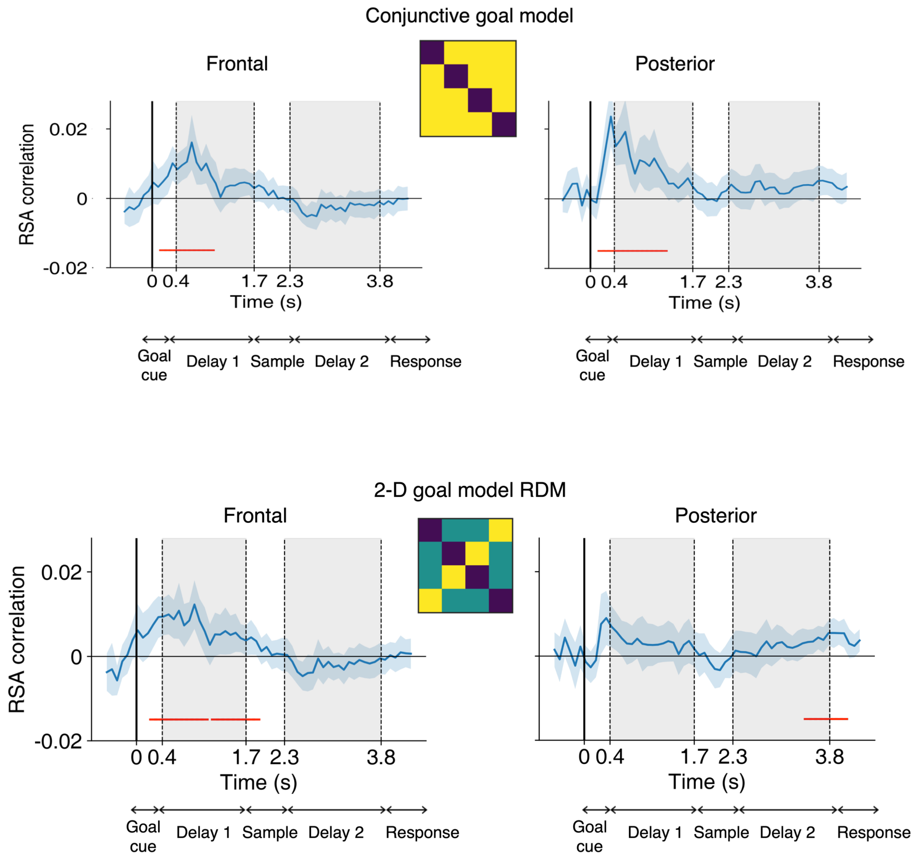

Response Figure 1. Time-resolved cross-validated RSA correlations using conjunctive (upper panel) and 2-D model (lower panel) with individual condition-averaged data in frontal and posterior channels, respectively. Data was averaged temporally within a non-overlapping 80-ms sliding window. Red horizontal lines denote significant time points (α = .05) corrected using a cluster-based permutation test. Error bars represent 95% confidence interval.

2. Related to the above, I'm particularly struck by the finding that posterior electrodes also showed a low-dimensional geometry (discussed in the second paragraph on p. 20). But earlier on, it was presented that sensory representations are thought to be high-dimensional, which seems to imply that different results would be found for frontal vs. posterior data. I can't quite wrap my head around this.

Responses: We apologize for not making this point clear. To clarify, we believe that the dimensionality of sensory representation could well depend on the nature of the stimuli, such as the number of relevant features. For example, when naturalistic objects/pictures were used, the dimensionality of the neural response can be high in the visual cortex (Stringer et al., 2019). By contrast, when structured stimuli were used (such as orientations or colors spanning a circular space), the representational geometry in the visual cortex can be reflective of that and hence low-dimensional, meaning that only two primary dimensions are needed to explain sufficient variance in

the neural data (Brouwer & Heeger, 2009; Li & Curtis, 2023). Given that our study artificially defined two stimulus axes and participants repeatedly learned about this space, it is possible that the stimulus representations reflected the color and size dimensions, with all the other irrelevant dimensions (e.g., stimulus category, texture, etc.) being compressed. We did not test the stimulus dimensionality in visual cortex prior to task learning, so whether stimulus dimensionality in our study was reduced after learning remains to be tested in future research. Nevertheless, it has been shown in previous studies that task demands can shape the dimensionality of neural responses. For instance, dimensionality in frontal cortex can be reduced due to task-based compression of neural code (Flesch et al., 2022; Mack et al., 2020). We have now updated the Discussion to make this point clearer (lines 543-557).

3. Method-wise, a somewhat odd choice of the analysis pertains to the way data were combined across subjects. It seems the data matrix are simply concatenated horizontally, which is analogous to running only a single subject with a lot more conditions, if I understand correctly. Is this correct? More explanation/citation of this method, and/or some simulation exercise, would help.

Responses: We indeed concatenated each subject's matrix horizontally, however, the number of conditions was not expanded but the number of features was (because the end product is n_condition * [n_feature * n_subject]; here feature refers to electrode in EEG and voxel in fMRI). This corresponds to a fixed-effect analysis which was also adopted in a recent study using a similar approach to visualize spatial working memory (Li & Curtis, 2023). Nevertheless, we agree that examining individual-level results for the geometry analysis is important for the validation of the effects (Reviewer 3 also raised a similar concern). In response to these comments, we have substantially edited this part of result in the revised manuscript, using a random-effect equivalent approach instead, which involves calculating circularity for each individual separately. The original fixed-effect, group-level results are still retained for visualizing the 2-D geometries in the revised manuscript. A summary of the changes can be found in response to Reviewer 3's Point 9; overall, our conclusions remain largely unchanged.

4. Regarding the analysis on frontomedial theta, Fig 4A shows that the majority of power in coherence is below 4Hz, so it seems odd to only use data from 4-7Hz, which is the nominal theta band, but it seems to miss the important stuff. Perhaps there is also frontomedial delta?

Responses: We thank the reviewer for pointing this out. Following the reviewer's suggestion, we repeated the correlation analyses using frontomedial delta (1-3 Hz) and the representational geometries, the results of which are summarized below in Response Table 1 (p-values were FDR-corrected). In general, the frontomedial delta (1-3 Hz) was not related to goal or stimulus-related circularities in any epoch of the task, suggesting that the relationship between EEG oscillatory activity and

representational geometry was specific to the theta band. We have added the delta band results to lines 324-326 in the revised manuscript and also to Supplementary Information (Table S1) to offer a more balanced view on the relation of EEG oscillatory activity and representational geometry.

		**Delay 1 theta**		**Delay 2 theta**		**Delay 1 delta**		**Delay 2 delta**	
		r	*p*	*r*	*p*	*r*	*p*	*r*	*p*
posterior goal circularity	Goal cue	-0.094	0.661	-0.321	0.925	-0.051	0.617	0.085	0.469
	Delay 1	-0.008	0.661	-0.118	0.925	0.187	0.478	0.317	0.280
	Sample	0.514	0.028*	0.449	0.049*	0.160	0.478	0.109	0.469
	Delay 2	0.439	0.040*	0.418	0.049*	-0.067	0.617	-0.156	0.757
posterior stimulus circularity	Goal cue	0.075	0.665	-0.054	0.538	0.344	0.941	0.143	0.737
	Delay 1	0.097	0.665	0.045	0.580	0.248	0.941	0.134	0.737
	Sample	-0.008	0.665,	-0.139	0.538	-0.042	0.850	-0.156	0.486
	Delay 2	-0.303	0.348	-0.545	0.016*	-0.276	0.424	-0.231	0.486
frontal response circularity	Goal cue	-0.222	0.840	-0.151	0.745	0.197	0.317	0.222	0.213
	Delay 1	-0.098	0.664	0.062	0.745	0.160	0.317	0.303	0.170
	Sample	-0.151	0.840	-0.132	0.745	0.019	0.467	0.134	0.276
	Delay 2	0.311	0.320	0.430	0.088	0.315	0.308	0.358	0.170
Posterior response circularity	Goal cue	0.377	0.114	0.146	0.455	0.381	0.160	0.178	0.354
	Delay 1	0.226	0.206	0.073	0.455	-0.009	0.510	-0.032	0.556
	Sample	0.345	0.114	0.300	0.348	0.0975	0.442	0.141	0.354
	Delay 2	0.179	0.212	0.025	0.455	0.147	0.442	0.140	0.354

Response Table 1. Correlation of theta and delta band coherence with 2-D geometries. All p-values were FDR-corrected. * denotes $p < 0.05$.

5. The fMRI GLM analysis modeled trial-wise individual event of interest, while the rest of the events were lumped together. This procedure was repeated for each event, to

arrive at estimates for each event for each trial. I'm not too sure about this method, especially given the timing of events are fixed across trials. Rule number 1 in event-related fMRI is to temporally jitter events to allow separation of BOLD response in GLM. Are the authors confident that their fMRI data allow estimate of individual events on single trials??

Responses: We thank the reviewer for raising this point. We agree that estimating the magnitude of delay activity with fMRI is not a trivial issue, and using a boxcar function with fixed durations has certain disadvantages. According to (Sreenivasan & D'Esposito, 2019), there are 3 main approaches to this problem: 1) Our original approach of using a boxcar function with fixed durations, which accurately estimates the magnitude of activity during the delay but is suboptimal at separating contributions of maintenance-related activity from those related to adjacent events such as encoding and response; 2) Fixed delays modelled as an impulse. This method can be done by placing a short impulse function at the middle of the delay period and convolving it with the hemodynamic response function. While this method can better separate the delay from other events, it hinders the accuracy of activity magnitude estimation; 3) Using variable delays, which requires planning at the start of data collection. This has the strengths of both previous approaches but complicates single-trial estimation (which is what we performed in the study in order to calculate circularity).

In light of this, we repeated the GLM single-trial estimation with the second impulse method and found that in comparison to both the group- and individual-level results using the originally estimated beta values, the goal- and stimulus-related circularity showed a highly similar pattern (Response Figure 2), suggesting that these two GLM methods were comparable in the scope of current analyses. We have now included the individual-level searchlight result using both estimation approaches on lines 363-372 in the revised manuscript.

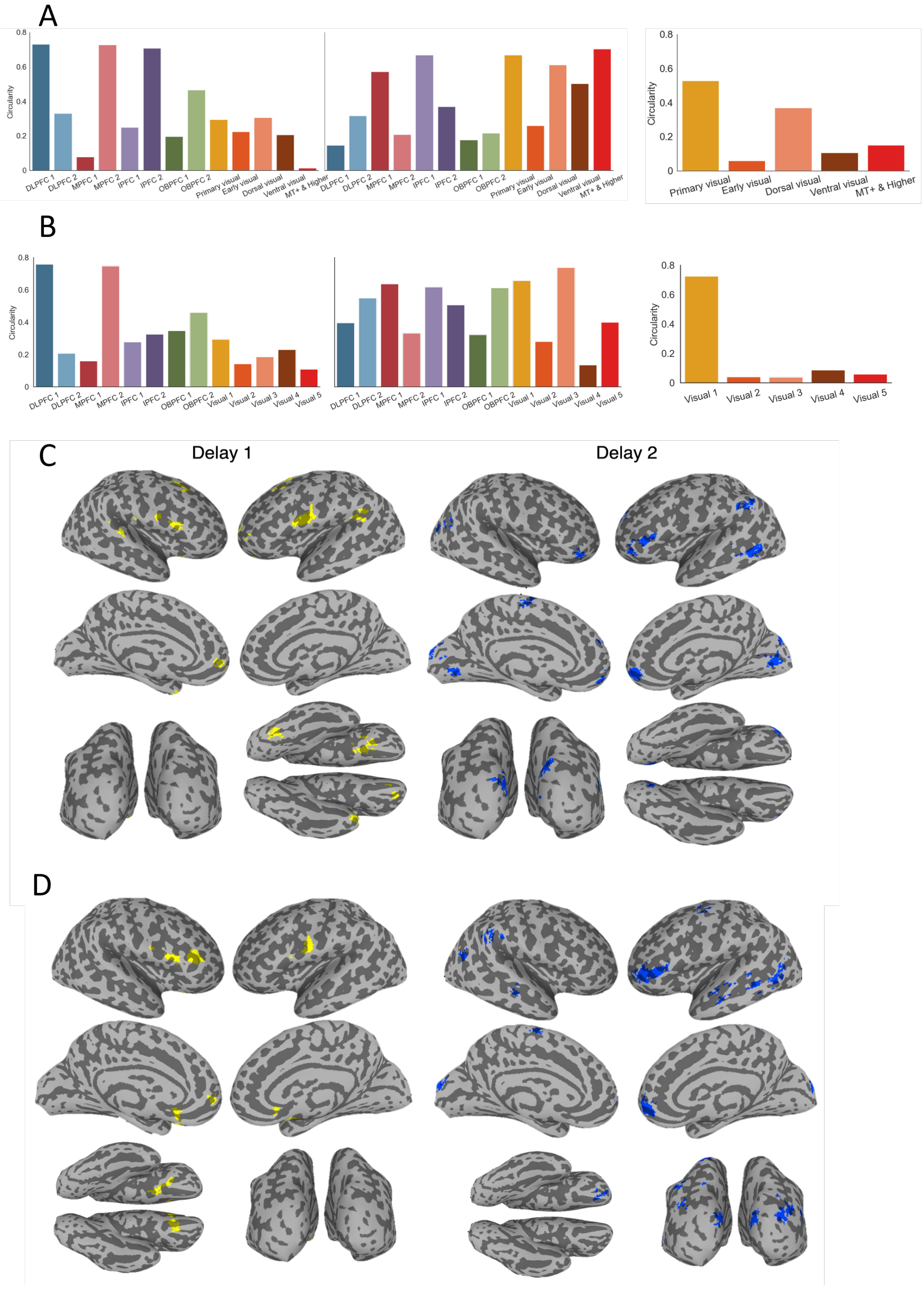

Response Figure 2. (A) ROI-based group-level goal circularity using delay activity estimated with an impulse response function. (B) Original result of group-level goal circularity using a boxcar function. (C) FMRI whole-brain searchlight result using an impulse response function. Significant ROIs show 2-D goal geometry in Delay 1 (yellow) and 2 (blue). (D) Same as (C) but using original beta estimates from a boxcar function.

6. The authors stated that the fMRI connectivity analysis showed "a robust bilateral

positive relationship" between prefrontal and dorsal visual ROIs (line 481-483) and referred to Fig 6B. However, this is not obvious at all from inspecting Fig 6B.

Responses: Indeed, the original sentence was not well structured and could cause misunderstanding. We intended to communicate that the prefrontal ROIs were positively correlated. In addition, both ipsilateral DLPFC 1 - dorsal visual ROI connections were also positive. The MPFC 2 - dorsal visual connection did not survive correction and therefore was excluded from discussion. We also want to take the chance to note that, in response to the reviewers' suggestions, we have updated all analyses using individual-level metrics instead of group-level results, and also replaced all ROI-based analyses with whole-brain searchlights. The functional connectivity analyses were then based on clusters identified in the searchlights. We demonstrated that in general the new findings are consistent with previous conclusion: the strength of functional connection between lateral frontal seed (seed 2 in Response figure 3), which coded 2-D task information in Delay 1, and early/dorsal visual areas (seed 3), which exhibited the same geometry in Delay 2, is predictive of memory performance (r = -0.56, p = 0.004) and shows a trend for goal circularity in the posterior seed (r = 0.32, p = 0.075). In contrast, such relationships did not exist when using the medial PFC cluster (seed 1) as the frontal seed (r = -0.19, p = 0.194 for correlation with response error; r = 0.16, p = 0.242 for correlation with goal geometry).

Due to changes in methodology, more clusters have been identified as representing 2-D goal geometry. Consequently, the connectivity analyses have adapted to a result-driven approach, focusing on the top 3 largest clusters identified in the whole-brain searchlights (see lines 420-435 for details). For this reason, some of the regions that were included in the ROI-based approach (e.g., medial PFC and visual areas) in the original manuscript are no longer reported in the revised manuscript, although they still showed significant 2-D goal representation according to the individual searchlight results.

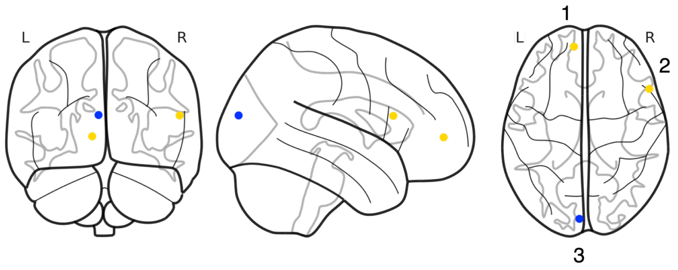

Response Figure 3. Location of the seed regions discussed in this point: Yellow denotes Delay 1 and blue denotes Delay 2.

7. This is a general comment. There are many results in this paper. However, there seem to be even more analyses that have been conducted. This is due to the richness of the data (e.g., different electrode groups, different events within a trial, different

neural and behavioral measures). The authors reported statistically significant results, which are probably only a small subset of all possible tests. This does leave one wonder if some of these results (especially correlations) could be accidental. Some control/correction for multiple statistical tests are needed. However, I don't find any mention for such correction. In general, I think the authors should give some assurance of the robustness of their results.

Responses: We apologize for the manner in which some results were reported in the original manuscript. We have made the following changes to provide more statistical rigor and the results are summarized below:

1. Whenever there were multiple comparisons, especially those happened for all task epochs, we have conducted FDR correction for multiple comparisons.

   These include the correlations between theta coherence and various circularity indices. Most conclusions were unchanged after correction (see response to Reviewer 2's Point 4 for details), except that the correlation between theta coherence and frontal response circularity became marginal ($p= 0.088$; Response Table 1). Another relevant section involves the correlations of fMRI functional connectivity with performance measures and circularity indices. Due to the fact that the definition of seed regions having shifted, the new results cannot be directly compared to the previous ones (see response to Reviewer 2's Point 6 for details). Nevertheless, the main finding that connections between frontal and posterior ROIs modulated goal- and stimulus-specific circularity, as well as correlated with performance remain unchanged with the new method and FDR corrections.

2. For the epoch-based (EEG) or ROI-based (fMRI) results that were now replaced by timeseries or by whole-brain results, respectively, we have instead conducted more suitable cluster-based permutation tests.

   In the EEG experiment, this point involves all the individual circularity time courses of goal, stimulus and response, as well as circularity difference between good and bad trials. Compared to the original epoch-based results, the critical periods when individual time courses show 2-D geometries were largely stable, except that frontal goal circularity now revealed an additional period of Goal cue (Figure 2B); and posterior stimulus circularity now only identified time points during Sample presentation (Figure 4A). For changes in split-trial analyses, posterior goal circularity only showed difference at Delay 2 (Figure 3C) and stimulus circularity did not show any difference over the entire trial (Figure 3D). In fMRI whole-brain analysis, we still observe that in Delay 1 clusters with 2-D goal representation were located in frontal cortex on both orbito-medial and lateral parts, whereas in Delay 2 most clusters centered in posterior regions, especially in the occipital and temporal cortices (Figure 6A). One distinction from previous finding is that in Delay 2 searchlight revealed some frontal regions, which may be a result of a combination of the shifts from using ROIs to whole-brain and from group- to individual-level

approach. However, it is noteworthy that goal geometry within these clusters was not mediated by connections with Delay 1 regions (Table S3), suggesting they may be separate from the process of relaying goal information.

3. We have also articulated more clearly the rationale of choosing some particular epochs for further correlation analysis, which was informed by significant results of previous steps.

   This point is relevant to Figure 3A, which showed correlation between goal circularity and behavioral performance; and Figure 4D, which showed behavioral relevance of theta coherence. Both analyses were motivated by results from previous steps that first identified significant time points of goal circularity or theta coherence. Hence, we did not test for other periods of the trial.

Reviewer #3: Using EEG and fMRI, this study investigates the representation of task and task specific information in the human brain during a goal-directed visual working memory task. It shows that a low-dimensional neural geometry of goal information first appears in frontal cortex and then transferred to posterior sensory cortex through theta coherence. Goal-directed stimulus processing is an important component of human cognition and has been studied by an extensive amount of prior work. Overall, I am unsure about the novelty of this study and how it may contribute to this vast literature beyond what we already know. The review of the literature is also missing some key information. Some of the assumptions and manipulations in the study are made without detailed justifications. I also have some questions regarding data analyses.

We thank the reviewer for the constructive comments, which we believe have helped us improve the manuscript substantially. Please see below for a detailed point-by-point response.

(1) I am surprised that there is not an extensive review of the existing literature regarding neural synchrony during task performance between frontal and posterior regions, especially from monkey neurophysiology, such as Salazar et al. (2012, Science) and Siegel et al. (2015, Science). This existing literature already shows that goal information forms in frontal cortex and controls stimulus processing in posterior cortex. As such, I am not sure the main conclusion of the present study is novel. There is also an extensive literature regarding task representation in frontal cortex (e.g., the extensive work from Badre et al.). It is unclear how the present study add to that body of work.

Responses: We thank the reviewer for the constructive suggestions. We have included a more thorough review of the literature in the introduction to better lay out the motivation for the current study. Although previous research, especially from the monkey neurophysiology literature, has demonstrated how frontoparietal and posterior cortices support implementation of task-based, top-down control, they differ from the

current study in several aspects. First, in some of these studies (Saalmann et al., 2007; Salazar et al., 2012), task representation was not examined directly. Instead, its effect was measured by how attention or working memory signals influence stimulus-specific inter-areal coherence (e.g., fronto-parietal or parieto-occipital) to infer how task information (in this case, attention or working memory) influences stimulus representation. Second, the seminal work by Badre and colleagues on the abstract task representation in frontal cortex has mostly used univariate contrasts between tasks to investigate neural signals relating to different levels of control (Badre & D'Esposito, 2007; Badre et al., 2010). As such, there lacks a systematic characterization of the task representations and the influence thereof on stimulus representations within a single task, which can be better characterized using multivariate methods in combination with connectivity analyses such as in our study. Third, many of the relevant studies on task control did not include a delay period in their paradigm, preventing dissociation of working memory-related activity from cue/stimulus-driven activity (this point also relates to the reviewer's Point 2, which we will elaborate on below).

Therefore, we believe that the key novelties of our study, building upon previous work, are as follows: first of all, we designed a structured 2-D task goal space to systematically investigate the corresponding task representation. This design enabled the characterization of the task goal representational geometry at the population level in different task epochs and different brain regions within a single paradigm. Next, we included a delay period for both goal maintenance and goal implementation periods, to dissociate working memory-related activity from the cue/stimulus-driven activity. And lastly, during the goal implementation stage, participants needed to manipulate the remembered stimulus representation to a transformed stimulus representation following the specific task goal, allowing us to examine how goal-related long-range interaction between brain regions modulates the dynamic coding of specific stimuli in working memory.

Moreover, we would like to note that although there are studies adopting multiple cues for the same task, thereby dissociating cue representation from task representation (Siegel et al., 2015), our designed 2-D goal space offers a complementary and informative approach to address this issue, as we demonstrate that some brain regions may encode goal (cue) information during goal cue and maintenance periods, but in a distinct representational format from our designed 2-D format (Response Figure 1). We have more clearly articulated these points in the revised manuscript.

(2) It is stated in the intro that "(y)et within working memory research, the mechanisms of how neural codes of abstract task information and specific stimulus contents collectively support goal-directed behavior remains an open question". What is special about working memory? Why can't we apply what we already know about goal-directed processing from the existing literature to working memory? Why should task information representation in working memory differ from other types of processing? I

found the motivation of the study to be weak and does not point to a concrete research question beyond rather vague general statement.

Responses: We thank the reviewer for the constructive comments. Cognitive control is often defined as the process in which humans adapt behavior to serve flexible task goals, and most cognitive control literature agrees that this process takes place in working memory (Badre et al., 2021; Miller & Cohen, 2001). However, as we discussed earlier, due to limitations in the experimental design, working memory-related activity often cannot be dissociated from the cue/stimulus-driven activity. This issue is not trivial, as the working memory literature has demonstrated a clear distinction between sensory versus mnemonic activity (Kwak & Curtis, 2022). Take the prefrontal cortex for example, many neurons exhibiting strong stimulus-specific selectivity during memory encoding show little sustained neuronal activity during the delay period, and vice versa. In fact, neurophysiological studies recording from prefrontal cortex propose a dynamic coding scheme for task types, based on findings that neural codes between sample/cue- and delay-periods do not generalize to each other (Stokes et al., 2013). Besides changes in the neuron tuning preferences, memory codes are also thought to be underlined by several alternative mechanisms, such as sub-threshold activity during delay compared to perception in sensory cortex and activity-silent mechanisms (Mongillo et al., 2008; Rose et al., 2016; Wolff et al., 2017). Considering these major distinctions, insights gained from the cognitive control literature that do not intentionally separate perceptual and mnemonic processes cannot be directly applied to the latter, although it could guide the investigations.

All the above discussion has been based on working memory studies for stimulus information, and it remains unclear whether working memory of abstract task goals also engages similar neural mechanisms. In parallel, we also note that a body of work demonstrates inter-areal theta-band coherence as a mechanism for working memory maintenance of stimulus information (Daume et al., 2017; Liebe et al., 2012), which also requires further investigation with goal representation in working memory.

By recruiting two delay periods in the task (Delay 1 for goal maintenance and Delay 2 for goal implementation), we were able to show the coding dynamics of goal information: the goal geometry was represented in an active neural code during goal maintenance in Delay 1 in frontal cortex, it shifted to more posterior sites during goal implementation in Delay 2. These results suggest rich dynamic coding schemes of goal information across different temporal epochs of the task and can only be unveiled through working memory paradigms that allow such development to take place. We have included a discussion on this issue as well as relevant results in the revised manuscript.

Altogether, we believe that our study provides (one of) the first attempts to unveil the neural coding mechanisms of abstract information in working memory and how this mechanism may collaborate with theta coherence to implement task goals on stimulus information.

(3) What is a low-dimensional neural subspace vs a high-dimensional one? How is it exactly defined? The task in the present study only involves a limited set of manipulations. So it is expected that the task structure would be low-dimension. Perhaps more explanations can be provided here. It is also presumed that task should form a two-dimensional structure in regions involved in task processing. What is the justification for this assumption? Task could be represented in an integrated manner in some brain regions rather than forming a two-dimensional structure, especially with learning. This possibility and its implications should be discussed.

Responses: We thank the reviewer for raising these points. In our view, the main advantage of having an experiment design with 2 orthogonal factors (task goals) is that it enables characterization of task-related representational geometry of goal information, beyond simple decoding of the presence of goal representation without specifying its representational format. However, although this 2-D representation aligns well with the task structure, it is not mutually exclusive with other formats of signal. In fact, other formats such as conjunctive representation could also exist in different brain regions or at different times of the task, but these signals may not reflect the task structure and could serve other purposes. To illustrate this, we performed an RSA analysis using a conjunctive model (which assumes that all goals are equidistant without forming a 2-D structure) and compared it with the 2-D model in the main text. Interestingly, we found that the conjunctive model was significant in the posterior channels during goal presentation and maintenance (Goal cue and Delay 1). By contrast, the 2-D model was significant during Delay 2 towards Response, consistent with our circularity analysis (Response Figure 1). These results suggest that sensory representation of goals is more likely to be in the conjunctive format, while the implementation of goals is more likely to be in the 2-D format, in line with our proposal that the 2-D geometry prompts goal transfer and implementation. We have made clearer in the revised manuscript (on lines 110-129; 175-177; 499-503) the reasoning of our experimental design. We also propose that future studies that include a new task with a different goal structure will add to a more comprehensive view of the goal geometry.

(4) I don't fully understand the circularity index. Has it been used in other published studies to document orthogonal representations? Why is it a valid quantitative way to characterize the geometry? Why not just calculate the vector angles and see if it is close to 90 degrees? Why would a larger circularity index indicate higher similarity between the two structures? More detailed descriptions and proofs would be very helpful here. If the geometry is a rectangle rather than a square (i.e., with the two dimensions forming less orthogonal representations), what would the circularity value be? Also chance level circularity of .74 is rather higher, and only slightly lower than the significance circularity of .77 reported. Why is chance level circularity so high? Why not just use the RSA measures reported in lines 238-248?

Responses: Circularity index was a metric originally adopted in (Li et al., 2020) to

quantify the geometric property of a perceptually uniform stimulus space based on perceptual similarity judgement data. The authors projected the data matrix to a 2D plane and measured the circularity formed by the projected coordinates of all conditions to assess data uniformity. It is rooted in a mathematical ratio between shape's area and square of perimeter, which reaches peak value when the shape has all equal sides (e.g. example 2, 5 and 7 in Response Figure 4), and decreases as it deviates from it. This serves our purpose of quantifying the representational geometries as we want to test for the specific two-dimensional space that assumes a square-like structure. To this end, while measuring angles between all pairs of vectors offers a similar solution to some extent, it cannot distinguish between regular rectangles and squares (as they all have 90-degree angles), and is also difficult to implement with more than four points (such as six/eight points for our stimulus geometry).

The magnitude of the circularity values depends on several factors, including the number of conditions and number of participants being averaged in the individual circularity analysis. For instance, with only four conditions it is relatively easy to achieve a high circularity value, whereas the value is reduced as the number of conditions increases to six (as in the case of stimulus circularity). However, in either case, changes in circularity are reflected in the null distribution as well. Because we shuffled trial labels to form a null distribution, selected the 95th percentile as the cut-off, and obtained the significance by comparing the actual circularity value against the null distribution - the significance remains valid despite changes in the absolute value of circularity. In addition, in response to the reviewer's Point 9, we have now replaced all group-level results with individually-calculated metrics. The shift to individual circularity also resulted in overall lower circularity values (because individual values were averaged across participants), but our interpretation of the significance remains valid as the null distributions were acquired differently as well.

RSA is also commonly used in assessing representational structure of multivariate data. In our study, we show that our circularity results are broadly in line with the RSA results, confirming the validity of our circularity measures (Response Figure 1). However, RSA differs from the circularity approach in several aspects. First, circularity analysis requires that the goal representation forms a specific task-relevant 2-D geometry, while 2-D RSA models only require increasing distance between goal representations as goal similarity decreases. As such, circularity analysis imposes a stricter restriction on the representational geometry. Relatedly, circularity analysis focuses on the neural geometry of the first two dimensions, while 2-D RSA models test all dimensions of the neural data. We believe that circularity analysis offers a complementary and intuitive perspective to understanding the neural representational structure of goal information, akin to how neural geometry analyses have contributed to understanding population-level representations in neurophysiological data in non-human primates.

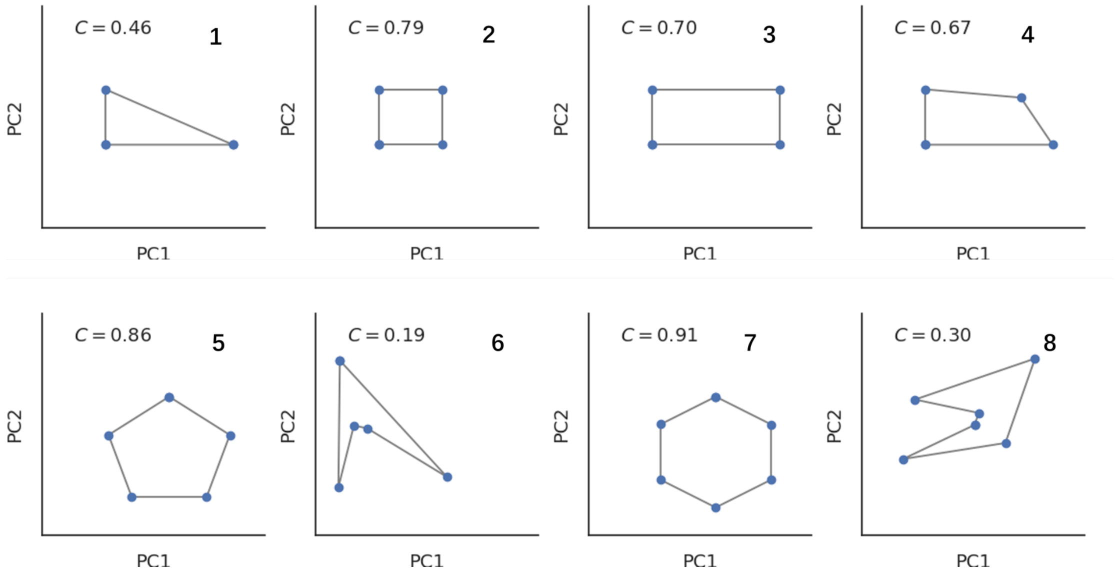

Response Figure 4. Simulations of circularity indices with various shapes and numbers of points.

(5) Could the behavioral correlation result reported in lines 250-262 simply due to participants forgot the goal in some trials and made the wrong adjustment? If so, this would not be a surprising result.

Responses: We thank the reviewer for raising this possibility. In response to the reviewer's point, we conducted a circularity analysis using only trials in which adjustment direction was incorrect. Specifically, we labeled the goal type based on participants' responses: if the direction of the response (redder, greener, bigger or smaller) was different from the cued goal, we characterized these trials as incorrect adjustment trials. If participants were holding the wrong goal in mind, then using the incorrect goal label should yield similar 2D geometry. However, this analysis showed no above-chance circularity at any time of the trial in either frontal or posterior channels, and this result was confirmed by both the group- and individual-level analyses (Response Figure 5). As such, we believe that remembering the wrong goal cannot account for the worse circularity results for incorrect trials. We have included this result to lines 236-238 of the revised manuscript, and also added a Supplemental Figure S3B.

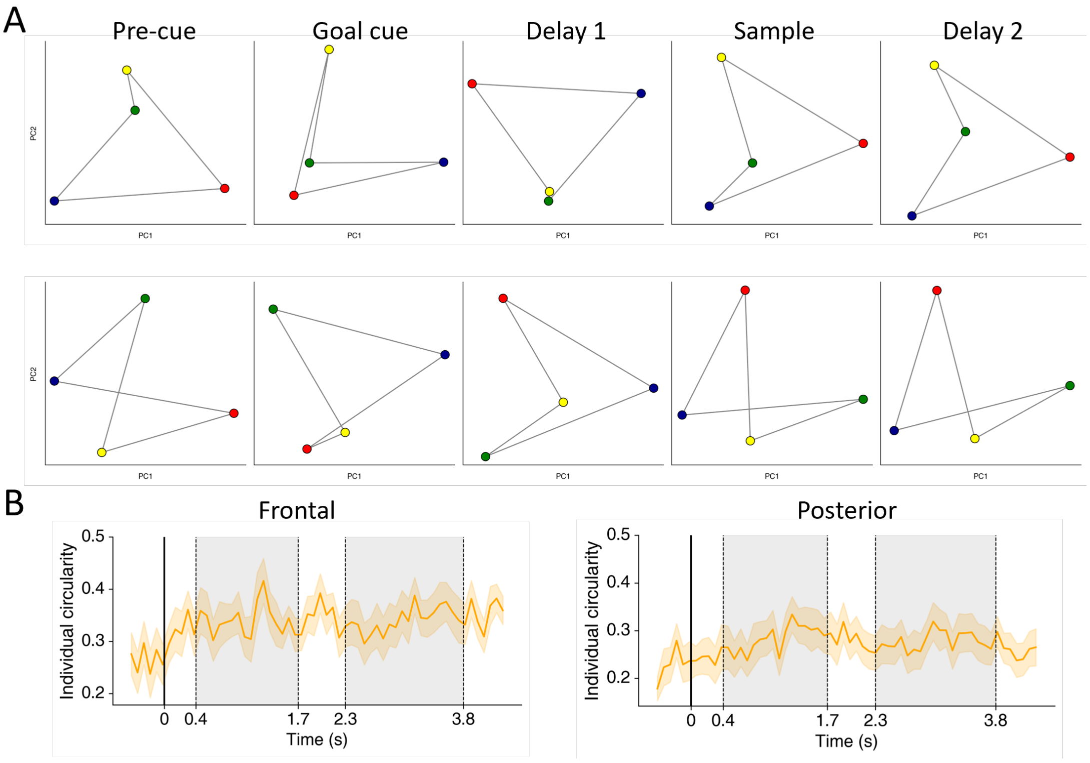

Response Figure 5. (A) Representational geometry of incorrect trials defined as those in which participants adjusted the sample stimuli to a different direction from the task goals, using group-concatenated data for frontal (upper) and posterior (lower) channels. (B) Time-resolved individual circularity of incorrect trials for frontal (left) and posterior (right) channels. Statistical significance was assessed by cluster-based permutation test compared to a null distribution.

(6) Is it valid to bootstrap the data to achieve a more stable estimation for the difference in 2-D geometry strength (line 269-270)? The whole point of statistical test is to compare the amount of effect with respect to the amount of noise. If noise is artistically reduced by bootstrapping, then the procedure basically artificially boost the strength of the effect.

Responses: We thank the reviewer for pointing this out. We agree with the reviewer and have revised the way we performed statistical analyses for circularity difference to use permutation testing. Permutation was performed by randomly swapping the trials between the large- and small-error sets and calculating the circularity difference to generate a null distribution. Compared with bootstrapping, it avoided the problem of artificially boosting the strength of the effect. Critically, the new results show largely comparable patterns to those reported before (Response Figure 6 and Figure 3 in main text). We are very grateful for this prompt – permutation-based approach is stricter, but indeed a more rigorous way to test the effect.

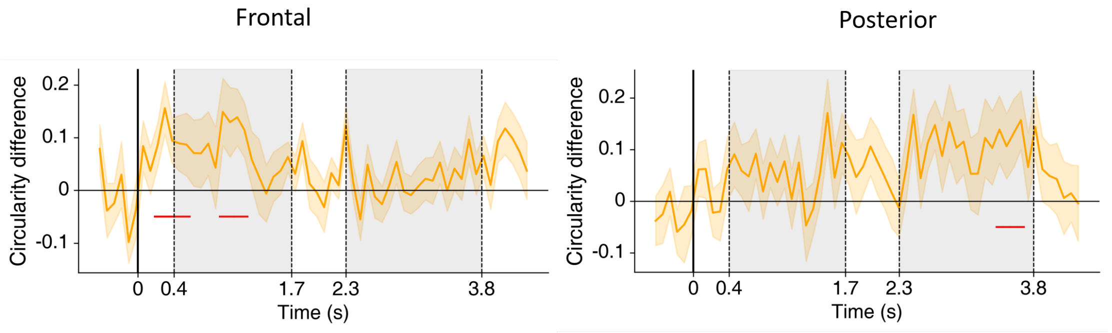

Response Figure 6. Time-resolved difference in frontal (left) and posterior (right) 2-D goal geometry between trials in the upper and lower quartiles based on response errors. Red horizontal lines denote significance time points at α = 0.05. Error bars denote SEM.

(7) It is argued that in line 301 that the significant result of stimulus geometry obtained in Delay 1 was likely due to sporadic fluctuations. This is rather post hoc. How do we know other significant results are not due to such sporadic fluctuations?

Responses: We agree with this point. Indeed, this contradiction has evaded explanation and motivated the switch to a more robust approach to assess the geometry. As touched on in our response to Point 9, we now switched to calculating individual circularity index and as a result, the accidentally high circularity values due to natural fluctuations was no longer present in the data. Changes have been made throughout, but relevant to this comment we show below that stimulus-related circularity is significant only during Sample (Response Figure 7), which in retrospect confirms that the result in Delay 1 in the original manuscript was possibly a sporadic phenomenon.

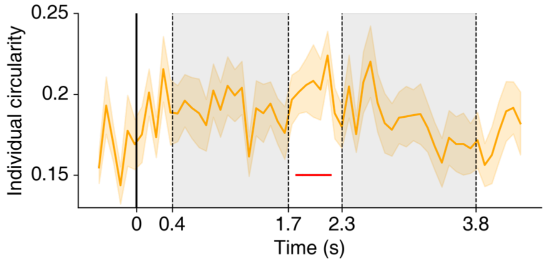

Response Figure 7. Time course of posterior individual circularity index using data averaged over a non-overlapping 80-ms sliding window. Error bars denote SEM. Red horizontal lines denote time points when 2-D geometry was significant in cluster-based permutation test compared to a null distribution (α = 0.05).

(8) I don't fully understand why the goal representation shown cannot reflect motor

related processing. It could still reflect abstract motor plan that is being formed before an actual motor response is executed.

Responses: Our logic is that if a 2D motor representation exist, it should be the strongest towards the Response period, as participants are getting ready to execute the plan. However, we did not observe such a pattern in our result. Moreover, in the revised manuscript we have re-calculated motor-related circularity using the individual, method as we elaborate in Point 9. We did not find any significant time point associated with 2D motor representation within either frontal or posterior channels throughout the trial time course (Response Figure 8). This result again confirmed that motor-related processing is unlikely to account for the goal geometry in our study.

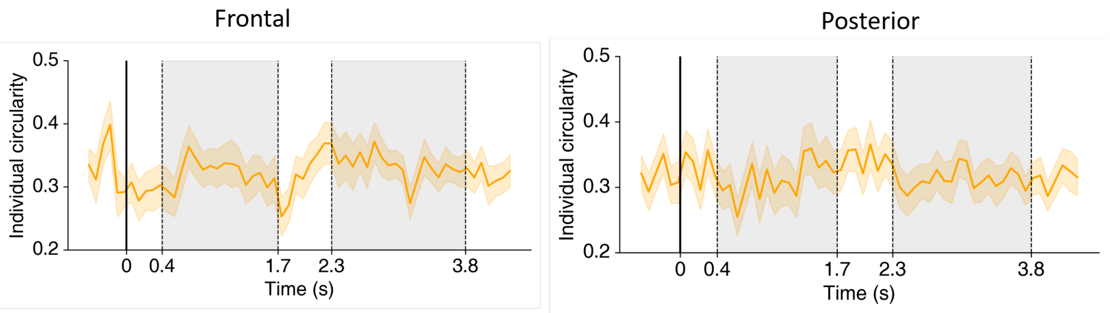

Response Figure 8. Individual circularity time courses of motor preparation signals for frontal (left) and posterior (right) channels. Error bar denotes SEM. No significant time points were found using a cluster-based permutation test.

(9) Group-concatenated data are subjected to the shortcomings of the fixed-effect analysis in that a few of the participants can drive the entire effect. Only results from random-effect analysis in which data are not concatenated should be reported.

Responses: We thank the reviewer for this constructive comment. Following the suggestion, we have updated our results with a random-effect equivalent approach (i.e., individual circularity) and only kept the group-concatenated ones for visualization purpose. The p-values for the group-level circularity were no longer computed. Statistical significance is assessed by acquiring null distributions via shuffling trial labels to which real data is compared. The improved method has led to similar conclusions in terms of where and when 2D geometry existed for EEG (Figure 2 and Response Figure 9). Specifically, time-resolved individual circularity analyses revealed that 2-D goal geometry existed during Goal cue and Delay 1 in frontal channels and in Delay 2 and Response in posterior channels.

For fMRI, this has prompted us to switch to a whole-brain searchlight approach to more thoroughly explore the localization of 2-D task representations. We observed that in Delay 1 most of the identified clusters were located in frontal cortex on both orbito-medial and lateral sides, whereas in Delay 2 most clusters aggregated in posterior

regions, especially the occipital and temporal cortices (Figure 6 and Response Figure 9). One distinction from previous finding is that in Delay 2 searchlight revealed some frontal regions, which may be a result of a combination of the shifts from using ROIs to whole-brain and from group- to individual-level approach. However, it is noteworthy that goal geometry within these clusters was not mediated by connections with Delay 1 regions (Table S3), suggesting they may be separate from the process of relaying goal information.

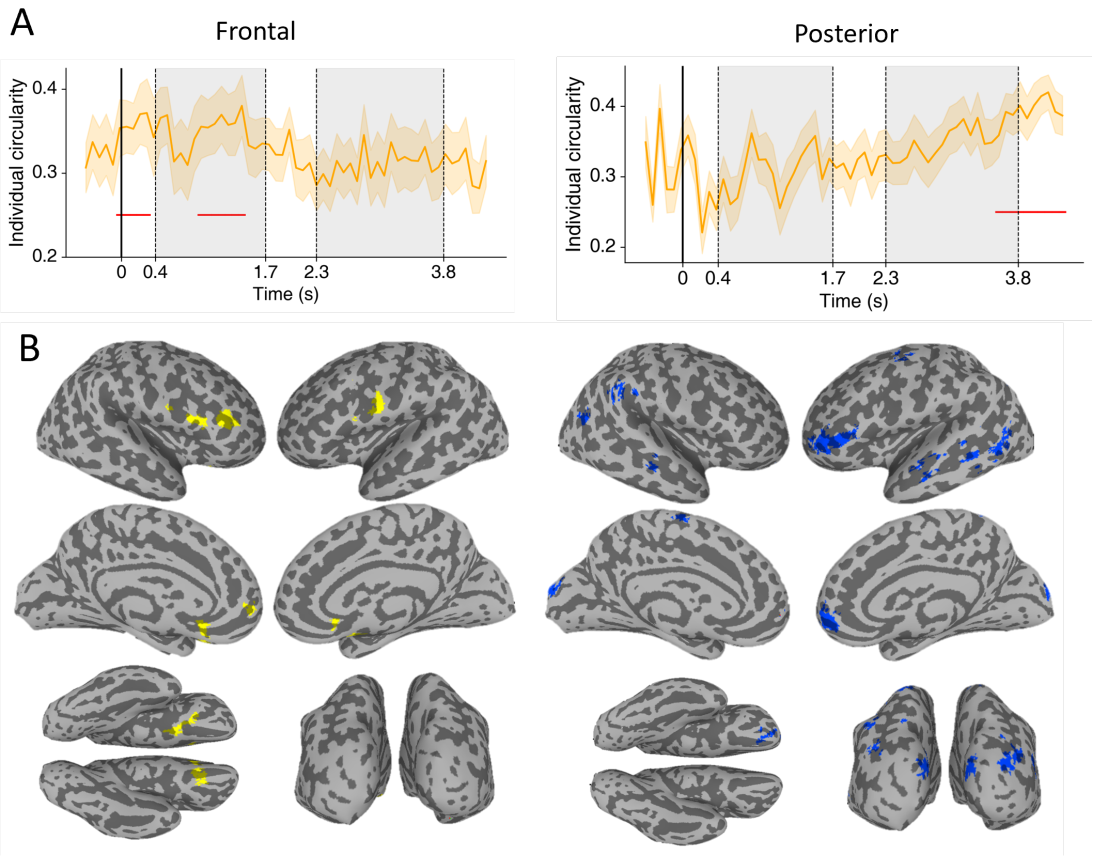

Response Figure 9. (A) Time courses of individual circularity index using data averaged over a non-overlapping 80-ms sliding window. Error bars denote standard error of the mean (SEM) using the number of participants as the degree of freedom. Red horizontal lines denote time points when 2-D goal geometry was significant in cluster-based permutation test compared to a null distribution ($\alpha = 0.05$). (B) FMRI whole-brain searchlight using individual circularity index. Significant ROIs with 2-D goal geometry in Delay 1 and 2 were shown in yellow and blue respectively.

(10) I am wondering how accurate EEG signal source localization is. Can we be sure that frontal sensors only have signals originate from frontal cortex?

<u>Responses</u>: This is indeed a valid concern. Throughout the manuscript we are very keen to avoid claiming spatial specificity from EEG results, precisely for the reason the reviewer highlighted. This in part has motivated the fMRI experiment as well. In

practice, researchers generally do not perform source reconstruction on 32- or 64-channel EEG as due to issues such as ill-conditioned covariance estimation, poor quality for deep brain source and lack of individual head models, etc. With these limitations in mind, we attempted to inverse the EEG data to a Freesurfer average brain space (Response Figure 10). We want to emphasize that while this method provides some insight into the spatial distribution of brain activity, it does not offer the level of precision afforded by fMRI. We found that frontal channel activity is generally localized to anterior section of the brain, so is posterior activity to the posterior section, and they do not appear to cross-contaminate. We have made some wording changes in the revised manuscript to be more rigorous in our interpretation of the EEG results.

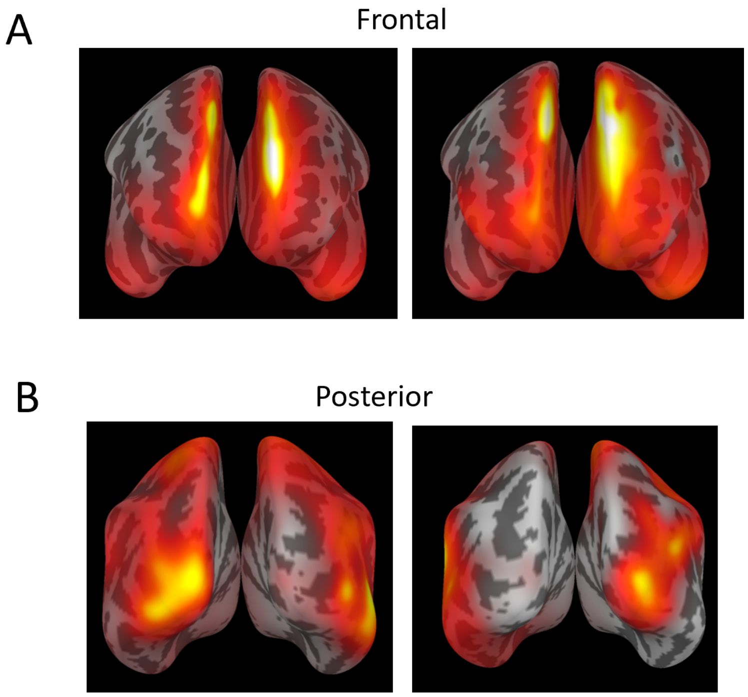

Response Figure 10. (A) EEG source reconstruction result for frontal channels activity in Delay 1 and 2. (B) EEG source reconstruction result for posterior channels activity in Goal cue and Sample.

(11) It took me a while to understand the task. Some more detailed descriptions and justifications of the task are needed. There are basically a fixed number of sizes and colors in a two-dimensional space. When participants are cued with stimulus X, they have to move one step in this stimulus space in the direction specified by the goal. This task requires long-term memory learning and retrieval of the stimulus space in addition to holding both task goals and stimuli in working memory. There was no motivation and justification for the task design. Why is this specific paradigm chosen?

Responses: We thank the reviewer for prompting us to explain more thoroughly the logic behind our task design. The purpose of the current study is to investigate how structured goal information is maintained and implemented in working memory. In

essence, participants need to mentally manipulate the stimulus information according to specific task goals. By having participant learn the stepwise-change in the stimulus space, we were able to dissociate the goal and stimulus representations, this means that for different stimuli, the same goal would lead to different correct responses. It is critical if we were to study their representations independently. To put it in another way, the stimulus space had to match the task space for the goals to apply upon. Another key component is setting a fixed degree of manipulation which requires learning, otherwise there would be no correct answers to compare responses to. In short, in order to investigate representational format of task information, some forms of structure have to be introduced to our paradigm, among them 2-D is one of the least complicated.

Regarding the task involving long-term memory, we note that classic working memory manipulation paradigms with a similar purpose also evoke it. For example, the mental rotation task (mentally rotating a remembered stimulus, such as spatial location, orientation, or object by a specific angle) is a typical manipulation task in working memory, where the rotation angle is acquired from long-term memory learning (Christophel et al., 2015; Kandemir et al., 2024; Shi & Yu, 2024). The only difference is that the rotation angle is acquired through prior knowledge (e.g., through prior math classes), while the steps in the stimulus space in our study are acquired during the study itself. Since we used a two-dimensional stimulus space to probe structured representations, we aimed to ensure comparability between the two stimulus axes, and therefore both stimulus axes were learned during the study, rather than relying on prior knowledge for one dimension (e.g., spatial location or orientation) to simplify the task. We have included relevant information in the revised manuscript to elucidate our reasoning.

References

Badre, D., Bhandari, A., Keglovits, H., & Kikumoto, A. (2021). The dimensionality of neural representations for control. *Curr Opin Behav Sci, 38*, 20-28. doi:10.1016/j.cobeha.2020.07.002

Badre, D., & D'Esposito, M. (2007). Functional magnetic resonance imaging evidence for a hierarchical organization of the prefrontal cortex. *J Cogn Neurosci, 19*(12), 2082-2099. doi:10.1162/jocn.2007.19.12.2082

Badre, D., Kayser, A. S., & D'Esposito, M. (2010). Frontal cortex and the discovery of abstract action rules. *Neuron, 66*(2), 315-326. doi:10.1016/j.neuron.2010.03.025

Brouwer, G. J., & Heeger, D. J. (2009). Decoding and reconstructing color from responses in human visual cortex. *J Neurosci, 29*(44), 13992-14003. doi:10.1523/JNEUROSCI.3577-09.2009

Christophel, T. B., Cichy, R. M., Hebart, M. N., & Haynes, J. D. (2015). Parietal and early visual cortices encode working memory content across mental transformations. *Neuroimage, 106*, 198-206. doi:10.1016/j.neuroimage.2014.11.018

Comeaux, P., Clark, K., & Noudoost, B. (2023). A recruitment through coherence theory of working memory. *Prog Neurobiol, 228*, 102491. doi:10.1016/j.pneurobio.2023.102491

Daume, J., Gruber, T., Engel, A. K., & Friese, U. (2017). Phase-Amplitude Coupling and Long-Range Phase Synchronization Reveal Frontotemporal Interactions during Visual Working Memory. *J Neurosci, 37*(2), 313-322. doi:10.1523/JNEUROSCI.2130-16.2016

Flesch, T., Juechems, K., Dumbalska, T., Saxe, A., & Summerfield, C. (2022). Orthogonal representations for robust context-dependent task performance in brains and neural networks. *Neuron, 110*(7), 1258-1270 e1211. doi:10.1016/j.neuron.2022.01.005

Kandemir, G., Wolff, M. J., Karabay, A., Stokes, M. G., Axmacher, N., & Akyürek, E. G. (2024). Concurrent maintenance of both veridical and transformed working memory representations within unique coding schemes. *Imaging Neuroscience, 2*, 1-20. doi:10.1162/imag_a_00173

Kwak, Y., & Curtis, C. E. (2022). Unveiling the abstract format of mnemonic representations. *Neuron, 110*(11), 1822-1828 e1825. doi:10.1016/j.neuron.2022.03.016

Li, A. Y., Liang, J. C., Lee, A. C. H., & Barense, M. D. (2020). The validated circular shape space: Quantifying the visual similarity of shape. *J Exp Psychol Gen, 149*(5), 949-966. doi:10.1037/xge0000693

Li, H. H., & Curtis, C. E. (2023). Neural population dynamics of human working memory. *Curr Biol, 33*(17), 3775-3784 e3774. doi:10.1016/j.cub.2023.07.067

Liebe, S., Hoerzer, G. M., Logothetis, N. K., & Rainer, G. (2012). Theta coupling between V4 and prefrontal cortex predicts visual short-term memory performance. *Nat Neurosci, 15*(3), 456-462, S451-452. doi:10.1038/nn.3038

Mack, M. L., Preston, A. R., & Love, B. C. (2020). Ventromedial prefrontal cortex compression during concept learning. *Nat Commun, 11*(1), 46. doi:10.1038/s41467-019-13930-8

Miller, E. K., & Cohen, J. D. (2001). An integrative theory of prefrontal cortex function. *Annu Rev Neurosci, 24*, 167-202. doi:10.1146/annurev.neuro.24.1.167

Mongillo, G., Barak, O., & Tsodyks, M. (2008). Synaptic theory of working memory. *Science, 319*(5869), 1543-1546. doi:10.1126/science.1150769

Rose, N. S., LaRocque, J. J., Riggall, A. C., Gosseries, O., Starrett, M. J., Meyering, E. E., & Postle, B. R. (2016). Reactivation of latent working memories with transcranial magnetic stimulation. *Science, 354*(6316), 1136-1139. doi:10.1126/science.aah7011

Saalmann, Y. B., Pigarev, I. N., & Vidyasagar, T. R. (2007). Neural mechanisms of visual attention: How top-down feedback highlights relevant locations. *Science, 316*(5831), 1612-1615. doi:10.1126/science.1139140

Salazar, R. F., Dotson, N. M., Bressler, S. L., & Gray, C. M. (2012). Content-specific fronto-parietal synchronization during visual working memory. *Science, 338*(6110), 1097-1100. doi:10.1126/science.1224000

Shi, D., & Yu, Q. (2024). Distinct neural signatures underlying information maintenance and manipulation in working memory. *Cereb Cortex, 34*(3). doi:10.1093/cercor/bhae063

Siegel, M., Buschman, T. J., & Miller, E. K. (2015). Cortical information flow during flexible sensorimotor decisions. *Science, 348*(6241), 1352-1355. doi:10.1126/science.aab0551

Sreenivasan, K. K., & D'Esposito, M. (2019). The what, where and how of delay activity. *Nat Rev Neurosci, 20*(8), 466-481. doi:10.1038/s41583-019-0176-7

Stokes, M. G., Kusunoki, M., Sigala, N., Nili, H., Gaffan, D., & Duncan, J. (2013). Dynamic coding for cognitive control in prefrontal cortex. *Neuron, 78*(2), 364-375. doi:10.1016/j.neuron.2013.01.039

Stringer, C., Pachitariu, M., Steinmetz, N., Carandini, M., & Harris, K. D. (2019). High-dimensional

geometry of population responses in visual cortex. *Nature, 571*(7765), 361-365. doi:10.1038/s41586-019-1346-5

Wolff, M. J., Jochim, J., Akyurek, E. G., & Stokes, M. G. (2017). Dynamic hidden states underlying working-memory-guided behavior. *Nat Neurosci, 20*(6), 864-871. doi:10.1038/nn.4546
