# Supplementary material for: The representation of abstract goals in working memory is supported by task-congruent neural geometry
Source: PLoS Biol. 2024 Dec 19;22(12):e3002461. doi: 10.1371/journal.pbio.3002461 (PMC11703074; doi:10.1371/journal.pbio.3002461)
Supplement: Attachment — Submitted filename: Response letter_R2_Final.docx [file pbio.3002461.s014.docx]

**Point-by-point response to PBIOLOGY-D-23-03141R2**

We would like to sincerely thank the reviewers for their insightful and constructive comments and suggestions. In the revised manuscript, we have included additional discussion and have edited the manuscript following the reviewers’ suggestions. All changes in the revised manuscript are highlighted in red. Below we have reprinted the reviewers’ comments in black, and inserted our point-by-point responses in blue.

Reviewer #1 (Runhao Lu): The authors have niced answered my questions. I'm happy with the current version to be published.

We want to thank Reviewer 1 for taking the time to help us improve the manuscript.

Reviewer #2: The authors have addressed most of my comments. I appreciate the effort they made in the revision. I think the manuscript has improved significantly.

However, I do feel that my first comment in the original review was not completely addressed, regarding the circularity of the interpretation, i.e., quoting from the original review, "the researchers designed a low-dimensional factorial experiment, then they looked for neural signals that reflects such geometry, then concluded the low-dimensional signal underlies the behavior." In many parts of the paper (e.g., title, abstract, parts of the discussion), there is an emphasis on low-dimensional representation. If I understand it correctly, this approach will find high-dimensional signal if the task is designed to have a higher dimension. It seems more appropriate to talk about something like "task-congruent neural geometry", instead of "low-dimensional neural geometry". I think the central story won't change, i.e., task-related signals arise in frontal areas first and then propagate to posterior areas, which is interesting in itself. What I'm a little confused is about the
absolute dimensionality of the representation (whether it's low or medium or high), which seems to be a secondary/orthogonal issue and cannot be pinned down by the current study. I think the authors need to de-emphasize the absolute dimensionality of the results, or further justify their rationale.

Lastly, in the spirit of open science and transparency, I would strongly encourage the authors to share their data and code on a public repository. Given the richness of the data, this would also benefit the field for others to explore this dataset for additional findings that might be interesting.

**Responses**: We are grateful for Reviewer 2’s constructive and insightful suggestions.

Regarding the first point, previously we have included discussions on the possibilities of alternative representational formats, and emphasized that our task design and analyses did not exclude any functional indications related to them. Nevertheless, we acknowledge that the use of the term “low-dimensional” in the current context may coincide with common discussions in the field regarding the low versus high dimensionality of neural representations. While the task structure in the current study is indeed low-dimensional, discussion on dimensionality would remain equivocal without the inclusion of an additional task with higher dimensionality. To this point, we fully agree that “task-congruent” is a less ambiguous term to convey the central message. We have edited the manuscript throughout to reflect this change.

We are committed to open science and upon publication of the manuscript both data and codes will be available on a selected public repository as permitted by relevant regulations.

Reviewer #3: The authors have done a good job addressing the majority of my comments. However, I still have some remaining concerns (one major) that dampen my enthusiasm for the study.

Major:

In the previous round of review, I commented that:

"It is also presumed that task should form a two-dimensional structure in regions involved in task processing. What is the justification for this assumption? Task could be represented in an integrated manner in some brain regions rather than forming a two-dimensional structure, especially with learning."

In the reply letter, the authors stated that:

"To illustrate this, we performed an RSA analysis using a conjunctive model (which assumes that all goals are equidistant without forming a 2-D structure) and compared it with the 2-D model in the main text. Interestingly, we found that the conjunctive model was significant in the posterior channels during goal presentation and maintenance (Goal cue and Delay 1). By contrast, the 2-D model was significant during Delay 2 towards Response, consistent with our circularity analysis (Response Figure 1). These results suggest that sensory representation of goals is more likely to be in the conjunctive format, while the implementation of goals is more likely to be in the 2-D format, in line with our proposal that the 2-D geometry prompts goal transfer and implementation."

However, I don't see any discussion of this result in the revised manuscript. The design of the present study created a low-dimensional task space. Although some brain regions show such a low-dimensional structure, since a conjunctive task structure is also possible as described by the authors, examining only the low-dimensional task structure and drawing conclusions solely from such results may not be justified. It basically says that only the low-dimensional goal representation matters and others do not. What is the basis for this approach and assumption?

**Responses**: We thank the reviewer for prompting us to further clarify the issue. We have added results related to conjunctive goal representations in supporting information (Figure S2 and S6) as well as included a dedicated paragraph in Discussion (lines 576-583) to state the scope and limitations of the current study. Overall, the analyses suggested the conjunctive/high-dimensional representation was likely related to sensory signals induced by visual cues in the EEG experiment and was less evident compared to the 2-D counterpart in the fMRI experiment, possibly because we replaced the shape cues with textual ones.

We would like to note that as Reviewer 2 have correctly pointed out, the term “task-congruent” may be more accurate as what we wanted to emphasize was a goal geometry that was congruent with our 2-D task structure. It is not our intention to exclude other formats of goal representations or to draw conclusions based solely on the 2-D analyses. As such, we have replaced the term “low-dimensional” with “task-congruent” throughout the majority of the manuscript to avoid this confusion, and have included the RSA conjunctive model results to offer a more balanced view of the matter.

Minor:

(1) PFC may simply represent abstract information that needs to be retained during WM delay, whether such information specifies a goal or not. In other words, PFC may not be representing goal information per se, but any abstract info that is relevant in a WM task. In the present study, such abstract info happens to correspond to the goal of the second phase of the study. It would be helpful to discuss this.

**Responses**: To clarify, we indeed defined task goals as a type of abstract task information which is in contrast to the low-level sensory information such as stimulus color and size. For instance, on lines 54-56 of Introduction, “Neural activity in frontal cortex has also been found to preferentially encode higher-order, abstract representations of task contingencies, contexts, and rules/goals.” On lines 505-507 of Discussion, “Frontal cortex has long been considered as a core region for processing abstract task information, with … successful decoding of a variety of abstract task information.”

Nevertheless, we agree with the reviewer that this point should be made more explicit. We have now added the following information to the manuscript on lines 84-87: “In the present study, we set out to directly test this hypothesis by tracking the emergence of abstract task representations in different cortical regions and investigating how they interacted with specific stimulus contents during human working memory. In particular, we focused on goal information, a well-established form of abstract task information.”

(2) On lines 239-241 and lines 479-480, the authors stated that the fidelity of the goal geometry in PFC was predictive of individual behavioral memory performance. This implies a causal connection between the two, which has not been established. Both the goal geometry in PFC and behavioral memory performance may be influenced by the overall level of attention, fatigue, motivation, etc., rather than geometry determining performance.

**Responses**: We thank the reviewer for noting this and we have changed to using “correlated” or “associated” to avoid confusions.
